# Four Misconceptions About Nonverbal Communication

**DOI:** 10.1177/17456916221148142

**Published:** 2023-02-15

**Authors:** Miles L. Patterson, Alan J. Fridlund, Carlos Crivelli

**Affiliations:** 1 Department of Psychological Sciences, University of Missouri–St. Louis; 2 Department of Psychological and Brain Sciences, University of California, Santa Barbara; 3 School of Applied Social Sciences, De Montfort University

**Keywords:** nonverbal communication, body language, personal space, emotion, deception, facial expression

## Abstract

Research and theory in nonverbal communication have made great advances toward understanding the patterns and functions of nonverbal behavior in social settings. Progress has been hindered, we argue, by presumptions about nonverbal behavior that follow from both received wisdom and faulty evidence. In this article, we document four persistent misconceptions about nonverbal communication—namely, that people communicate using decodable body language; that they have a stable personal space by which they regulate contact with others; that they express emotion using universal, evolved, iconic, categorical facial expressions; and that they can deceive and detect deception, using dependable telltale clues. We show how these misconceptions permeate research as well as the practices of popular behavior experts, with consequences that extend from intimate relationships to the boardroom and courtroom and even to the arena of international security. Notwithstanding these misconceptions, existing frameworks of nonverbal communication are being challenged by more comprehensive systems approaches and by virtual technologies that ambiguate the roles and identities of interactants and the contexts of interaction.

Philosophers and scientists have long speculated about the meaning and impact of nonverbal behavior (for a review, see Knapp, 2006). In literature going back to Homer, descriptions of characters’ appearance and behavior were central for understanding individuals and their interactions. It has been 150 years since Darwin’s (1872) work, *The Expression of the Emotions in Man and Animals*, awakened scientific interest in nonverbal behavior, yet systematic research on the topic took nearly a century, with rapid growth beginning in the 1950s and 1960s. Helping to launch research on nonverbal communication (NVC) were publications in anthropology (Birdwhistell, 1952, 1970; E. T. Hall, 1959, 1966), sociology (Goffman, 1963, 1971), and psychology (Ekman, 1965; Sommer, 1961, 1969). Since those early works the field has mushroomed, and there are now hundreds of scholarly books and tens of thousands of journal articles on various aspects of NVC. In the last 20 years alone, three comprehensive handbooks (J. A. Hall & Knapp, 2013; Manusov & Patterson, 2006; Matsumoto et al., 2016) have provided in-depth reviews of the field.

Decades of research have yielded a wealth of data and theorizing regarding the forms and functions of NVC. Not all information is equal, though, and as in all science, the signal-to-noise ratio is imperfect. Some of what are considered abiding truths about NVC have endured despite disconfirming evidence and trenchant criticism. These “truths” have taken on mythlike status as a kind of received wisdom impervious to evidence, and so they endure as pseudoscience (e.g., see Denault et al., 2020, for a critique of pseudoscientific practices in the fields of security and jurisprudence; and see Krumhuber & Kappas, 2022, on the supposed authenticity of Duchenne smiles). Barrett (2019), writing broadly about psychology, used economist Paul Krugman’s term *zombie ideas* to describe these walking dead of the psychological landscape, notions that survive more by inertia than merit. Although they may preferentially plague the general public, they have propagated to businesses, artificial intelligence start-ups, national security agencies, public-safety officials, basic science researchers, and university lecturers worldwide.

The goal of this article is to examine critically what we consider four central misconceptions about NVC—namely, that people communicate using body language; that they have a stable personal space; that they use universal, evolved, iconic, categorical facial displays to express underlying emotions; and that they give off, and can detect, dependable telltale clues to deception.

We suggest, with all compassion, that these zombie ideas should be put out of their misery. In doing so, however, we also recognize more broadly the progress made in establishing NVC research on firm scientific ground. This is manifested in important findings on topics such as social skills, person memory, first impressions, interpersonal influence, bias, robotics, empathy, and psychopathology, to name a few (Ambady & Rosenthal, 1992; Esposito & Jain, 2016; Knapp et al., 2012; Manusov & Patterson, 2006; Philippot et al., 2003; Sternberg & Kostić, 2020). We focus on these four misconceptions in order to acquaint readers outside the area of study, be they researchers, policymakers, students, or the general public, with what is known and what is either unknown or unsupported. We hope as well that this article will be a useful guide for investigators who may wish to enter the area and advance current knowledge. In doing so, we suggest a systems approach to NVC that avoids these misconceptions and grounds research in the nuances of interpersonal interactions as they occur in everyday life, in diverse societies, and in the virtual landscape of the metaverse.

## Misconception No. 1: We Communicate Using Body Language

Public fascination with body language began shortly after the publication of Julius Fast’s (1970) popular book of the same name. In general, *body language* refers to one or more nonverbal behaviors (e.g., distance, posture, gaze, touch, facial expression) that presumably signaled something about individuals’ personality traits, relationships, motives, momentary states, and ultimately their veracity. It was frequently assumed that people’s body language, though indirect, conveyed more truth about them than their words did. By 1980, commentators, consultants, and media experts were routinely noting the body language of politicians, Hollywood celebrities, and others prominent in media. Televised sports announcers began commenting on players’ moods and attitudes during the games, with self-assured pronouncements that “you can see it in their body language.”

The body-language industry has proliferated with the recent explosion of social media. Countless popular books, seminars, and videos now link astuteness about body language to love, wealth, and fame. Media consultants not only write books but routinely offer training in how people can attain winning body language on camera, whether in TV interviews or on YouTube. Sales training seminars include nonverbal behavior as part of “perfecting your pitch” and “upselling the customer.” Relationship coaches have clients practice smiling, tone of voice, and posture to impress dating partners. Romantic partners are encouraged to learn each other’s “love language” (Chapman, 1992). Parents are taught how to use their nonverbal behavior to discipline their children with firmness but not cruelty. And, as we review later, the U.S. Transportation Security Agency (TSA) trains many of its airport agents to spot certain body movements on the theory that such movements can predict a possible act of terrorism.

Apart from whether these attempts at marketing and deploying body-language training have merit, we must first ask whether the term “body language” itself has merit. Like language, nonverbal behaviors inform and influence others. As we shall see, however, casting nonverbal behaviors as instances of a body language is misleading, because they lack some key features of formal languages: (a) propositionality, (b) a vocabulary, and (c) syntax.^1^ When we detail these features, it becomes clear how nonverbal behaviors are different.^2^

### Some key features of formal language versus body language

First, in contrast to nonverbal behavior, formal language is propositional, so it can transmit content that includes truth claims (i.e., self-standing statements that admit to verification). For example, in oral and written language, one can make assertions about various states of affairs that can be proven true or false (e.g., you have green eyes and brown hair, it is sunny outside, and today is your birthday). But what can one do with most nonverbal behaviors (e.g., you are on the receiving end of a smile, along with prolonged gaze, close approach, and physical contact)? We can ask what led to these behaviors and speculate upon what might happen next, but all the rest is guesswork. “I think they must really like me” might be accurate in a romantic context, but not in a boxing ring or on a used-car lot. Verifying inferences takes other evidence (see Lycan, 2019).

Next, in verbal language, words have fairly invariant meanings, and the set of vocabulary words within any culture’s language constitutes that language’s lexicon. The English word *dog* typically signifies domesticated mammals that walk on four legs and bark.^3^ Often, nonverbal behaviors are not so easily interpretable. If we ask a friend about the weather outside and she scowls, her expression may mean that (a) it is lousy outside; (b) it is so lousy outside that it is ridiculous that we asked; (c) she has far more important things on her mind than the weather; or (d) she is still upset from the argument we had yesterday and the last thing she wants to do is talk to us, especially about the weather. Deciphering her scowl requires further information, which could come from her gaze direction, posture, body orientation, interpersonal distance, and the presence (or absence) of touch; any of these, or a combination, might do the trick, but maybe not. Thus, compared with language, there is imprecision in nonverbal behavior because it lacks a vocabulary.

NVC also lacks yet another basic property of formal languages—*syntax*. Languages have rules about how words should be assembled to make them mean things. Order and context are crucial. For instance, “the bear killed the man” differs from “the man killed the bear,” a difference the man is likely to notice. Context is also crucial. The meaning of “I do not know how to address that” depends on whether one is handling a criticism or working at a post office. Thus, the propositionality of language, along with the complexity afforded by vocabulary and syntax, all make language immensely precise, useful, flexible, and efficient.

Studies of cerebral hemispheric laterality and specialization using electroencephalography and neuroimaging also suggest a contrast between language and NVC. People engaged in verbal tasks tend to show increased overall activity in the left hemisphere, whereas people engaged in nonverbal tasks showing increased activity on the right (Corballis, 2014). The same left-hemisphere predominance for spoken language appears to generalize to manual languages such as American Sign Language. Specifically, Broca’s area in the left frontal lobe looks especially active when people are speaking or signing, and Wernicke’s area at the left temporoparietal junction looks especially active when people are listening to speech or receiving sign language (Hickok et al., 2001).^4^ It seems from the neurology that the operative contrast is between language, whether spoken or unspoken, and nonlinguistic communication by nonverbal behavior.

### The power of the situation

The body-language concept also neglects the critical role of the proximate physical environment in NVC. The immediate physical setting affects the give-and-take of NVC in both direct and indirect ways (Oishi & Graham, 2010; Patterson, 2019; Patterson & Quadflieg, 2016). An obvious example of the direct influence of the setting is the design and arrangement of furniture. In an office setting, a common arrangement involves a visitor’s chair situated directly opposite and across the desk from the supervisor. The directly opposing arrangement, often with the supervisor’s chair higher than the visitor’s, signals greater power and dominance than an adjacent arrangement (Burgoon & Dunbar, 2006). Of course, the arrangement of furniture is only one feature of the environment that affects the quality and course of NVC. Lighting, room color, wall art, desktop artifacts, ambient temperature, the presence or absence of windows, the acoustic environment, and scents can also affect moods and interaction patterns (Patterson & Quadflieg, 2016). Despite the large variability of settings and all the potential combinations among their constituents, these behavior settings often provide a mnemonic function that cues and constrains the types of behaviors that individuals exhibit (Rapoport, 1982). In sum, the power of the situation greatly defines the elements of the interaction and their interplay.

These various setting features are sometimes deliberately chosen or manipulated by users. In such cases, these features may suggest something about users’ personality, attitudes, and goals (Gosling et al., 2008; Webb et al., 1966). In turn, astute visitors might use this feature information strategically to adapt their communication patterns to achieve their own interaction goals. In other cases, users may “inherit” a particular setting and simply adapt to its features. Of course, fixed-featured elements (e.g., the size of the room, the location of doors and hallways) are not easily manipulated after construction, but they are often designed to mitigate or to enable behaviors and modes of influence (Altman & Chemers, 1980). For example, social norms among Trobriand Islanders may prevent commoners from building houses or sitting on verandas situated higher than their chief’s, as a way to preserve customs of rank and status (Malinowski, 1922).

The indirect effects of the environment are evident before interaction commences. People rarely find themselves in a specific setting by accident; personal preferences, attitudes, abilities, and goals all affect their choices of particular environments. Nor does everyone have equal access to all settings. Despite the authors’ wishes to be rock stars, sports celebrities, and corporate CEOs, we still await the invitations and accolades. To put it succinctly, people select settings, and settings select people (Barker, 1968; Wicker, 1979).

The combination of self- and setting selection also increases the likelihood that, within any given setting, interactants will be more homogeneous and less diverse. This increased homogeneity facilitates the efficiency of interactions and increases the probability of behavioral concordance and, sometimes, clear mimicry among partners (Patterson, 2019). Furthermore, once in a particular setting, people’s behavioral options are constrained not only by the physical environment, but also by that setting’s social norms. In such circumstances, patterns of NVC owe more to the physical features and social norms of the behavior setting than to any supposed codes of body language.

With today’s technology, the features of settings no longer need to be physical. Zoom callers in their offices or kitchens routinely green-screen themselves in front of Hawaiian or Parisian backdrops, and TV anchors in comfy studios insert themselves electronically before centers of government and at global-conflict hot spots. Inhabitants of the metaverse navigate virtual worlds in which their appearances, social roles, and situational contexts are all fluid. Just as multilingual speakers code-switch, it is likely that many people will learn to “world-switch” in behavior, manner of self-presentation, and accommodation to the frangible settings features of multiple virtual worlds. Already, journals such as *Metaverse Creativity*, *Electronic Imaging*, and the *Journal of Virtual Worlds Research* are featuring articles that explore issues of identity and role in virtual worlds that possess synthetic features and synthetic physics, worlds in which people can fly and their bodies can freely merge, and in which the usual modes and mores of social conduct may not apply.

### NVC elaborated

If NVC does not qualify as body language, what is it? Different definitions of NVC emerged in the 1960s and 1970s as research burgeoned (MacKay, 1972; Watzlawick & Beavin, 1967; Wiener et al., 1972; for a review, see Patterson, 1983). Our approach here defines “nonverbal communication” as the sending or receiving of information (sometimes both) among interactants by virtue of their physical environment, appearance, and nonverbal behavior (Patterson, 2011). NVC can be both complementary to language and apart from it, whether it transpires in person or via electronic mediation. Besides these core differences between NVC and language discussed earlier, there are three important contrasts in how the two function (Patterson, 2011). First, whereas verbal language appears in utterances, NVC is always “on,” and can occur as long as there are cues from the current setting and the appearance and behavior of others. This can happen when people navigate their environments with no intention of interacting with anyone. People routinely communicate when they silently pass on a sidewalk, wait in line at a grocery store, or take a seat at a doctor’s office. These situations are what Goffman (1971) called “unfocused interactions,” in contrast to “focused interactions” that are organized around conversation. Second, in conversation, the speaker and listener roles usually alternate. In contrast, in nonverbal interactions, individuals can send and receive appearance and behavioral cues concurrently. Whether at a job interview, having coffee with a friend, or simply walking down the sidewalk, there is an ongoing reciprocal exchange of nonverbal cues (Patterson, 1995, 2019).

Finally, NVC often proceeds outside of awareness. The simple perception of a partner’s behavior is often sufficient to lead us to respond without realizing we are doing so. One conversation partner may lean forward and the other will lean back, only to have the two reverse positions a moment later. Both may be oblivious to their postural “dance” (Wheeler & DeMarree, 2009). In social settings, people certainly can remind themselves to monitor how they are moving their faces, changing their tone of voice, or adjusting their posture, but this is usually awkward and unnecessary. They manage to issue the nonverbal behavior that fits their attitude and intentions, both in conversation and in silently sharing common spaces with others (Patterson, 2008). Such nonverbal behavior can be lagged and reciprocal, as when people react to others’ comments or behavior. It can also be concurrent, as when people nod, flash their brows, or purse their lips at what the other is saying. Even “motor mimicry” (e.g., wincing at another’s injury) appears to be a dialogical act by which “I show how you feel” (Bavelas, 2007; Bavelas et al., 1986, 1988) and which can facilitate liking between partners (Lakin, 2013). This concurrent sending and receiving in NVC makes for an efficient streamlining of social interactions.

### Misconception No. 1: Conclusion

If “body language” were only a benign metaphor, the term would simply be misleading. As it is, what the term connotes is actually damaging. Designating NVC a language wrongly implies a relatively invariant rule book by which to decode specific appearance cues and behaviors. This errant assumption has fostered a multimillion-dollar body-language industry in books, seminars, videos, and other media, dedicated to revealing the hidden codes and subtle strategies that are promised to insure success in every aspect of social life. This snake oil may benefit some people by making them more comfortable and confident in social settings, just as placebos often help people feel better. Still, consumers should be wary of claims that they can purchase access to any secret language of the body.

None of this implies that the divinations of body-language commentators are always incorrect, but such claims are almost always oversimplified, evaluated in hindsight, and riddled with confirmation bias (Nickerson, 1998). The meaning and impact of specific behavior patterns are contingent upon a number of factors, including the setting features, the characteristics of the individuals involved (e.g., culture, gender, personality, attitudes), and the particular goals of the individuals (Crivelli & Fridlund, 2018; Patterson, 1983, 2019; Russell, 2017). Indeed, the broad context for a particular nonverbal sequence is always pivotal in determining both its inferred meaning and its impact.

## Misconception No. 2: People Have a Stable Personal Space

Everyone has experienced times when strangers, and sometimes friends and acquaintances, are just too close for comfort, leading us to draw back as they grow closer. These uncomfortable situations are often labeled *violations of personal space*, and it has been a topic of study for over a century. Simmel (1908) spoke of the “sociality of space,” declaring that there was a “geometry of social life.” In the 1920s, Bogardus developed his Social Distance Scale, believing that interpersonal distance reflected prejudice (Bogardus, 1925, 1926). Bogardus’s scale, with various revisions, was used widely in the study of racial and class prejudices and spawned a wave of applied social-distance research from the 1920s to the 1950s (Ethington, 1997).

E. T. Hall (1966) coined the term *proxemics* for the study of how space was used by members of different societies (e.g., Germany, Japan, and the Middle East), and took ideas that had been sociometric and extended them to interpersonal communication. Hall’s “distance zones” were spaces in which interaction occurred, not spaces surrounding individuals. It was Robert Sommer, an early researcher on spatial behavior, who proposed that social interactions were regulated by a personal space, which was “an area with invisible boundaries surrounding a person’s body into which intruders may not come” (Sommer, 1969, p. 26). The sociologist Erving Goffman described a similar boundary space and observed that violations of it engendered displeasure and sometimes withdrawal (Goffman, 1971, p. 30).

One indication of the popularity of the topic lies in the results of a PsycINFO database search for publications related to “personal space.”^5^ From 1970 to 2020, there were 1,798 peer-reviewed journal articles and book chapters, 294 dissertations, and 145 books on personal space. The search revealed a peak throughout the 1990s, a dip during the 2000s, and a powerful resurgence during the 2010s. Interest in personal space continues today, not only in numerous scholarly articles and chapters, but also in a variety of popular printed and online outlets coaching people on how to deal with those who do not “give us our space.” In addition, TV commercials feature such violations at the doctor’s office or in our cars, and public service announcements have called out people who did not comply with mandated COVID-19 social distances.^6^

### Personal space versus interpersonal distance

We first need to clarify two terms. Our personal space, as the term is commonly used, is what lies inside an invisible, stable, subjective boundary that we claim as ours, defend from intruders, and use to regulate our interactions. This notion does not capture the complexities of how we use space and respond spatially to others, as indicated by studies of *interpersonal distance*, which involve the objective, measurable physical separations among individuals in social settings. These studies show that preferred measured distances depend on the culture, gender, and personalities of interactants (e.g., E. T. Hall, 1966; J. A. Hall & Gunnery, 2013; Matsumoto et al., 2016; Patterson, 1978).

Another research approach manipulates interpersonal distances by seating arrangement or confederate behavior to examine the impact on other aspects of social interaction. For example, the consequences of spatial intrusions can be explored by measuring the reactions of unsuspecting participants to close approaches by experimental confederates (Brodsky et al., 1999; Felipe & Sommer, 1966; Patterson et al., 1971). Although interpersonal distance is objectively measurable, personal space is not. Again, personal space is subjective, defined, and verifiable only post hoc by measurements of people’s reactions to its invasion.

### Problems with personal space

More than 45 years ago, the first author issued a cautionary note that was critical of the construct (Patterson, “Personal space: Time to burst the bubble?,” 1975). In a review of the early personal-space research, Hayduk (1983) noted that the analogy of personal space as a bubble was too simplistic, and instead suggested that it might be considered a kind of three-dimensional force field where force decreases with distance. Regardless of the appropriate analogy, the problems with the personal-space construct remain the same as they were in 1975. First, the notion of personal space implies an invariant, stable boundary separation from others. The presumed stability of this boundary is belied by the fact that people allow friends closer than strangers and their children closer than their friends. With romantic partners, the preference is often no space at all. Even with the same interaction partners, we are likely to prefer greater separations at work than at a party or when the other is in a foul mood. Although we certainly grant that some people are more standoffish than others, the reality is that our spacing preferences shrink and expand moment to moment, depending on a variety of factors. How can we know which one of these momentary preferences reflects the actual personal space?

Second, managing space in social settings is interpersonal rather than personal. Spatial preferences usually are not attached to specific other people per se but to the social roles of the interactants and the context of the interaction.^7^ In seated interactions, the distancing options may be limited by chair placement and the social density of the setting. Standing interactions, however, allow for more fluid negotiations of initial spacing, with some dominolike rejiggering that resembles new birds joining their flock on an electric line.

Other behavioral adjustments can occur in ways that leave interpersonal distance unchanged. People sitting down next to us when other seats are free may lead us to avert gaze, maintain silence, and turn away from the intruder. Such compensation for the intrusion does not increase physical spacing, but it certainly increases psychological distance, and it mitigates the negative impact of the close approach (Patterson, 1973). In contrast to pushy strangers, comparably close approaches from close friends, children, and partners can precipitate reciprocation of their close approach, in the form of increased gaze, smiling, touch, and chatter (Patterson, 1976).

That people may either reciprocate or compensate for intrusions presents a third problem with personal space, namely that it fails to capture the complex, dynamic relationships among nonverbal behaviors when interpersonal closeness is negotiated. Everyday examples show how distance from others can be irrelevant to the overall impact of others’ presence. Would you rather have a generic stranger in an airport sit just beside you, or two seats away with an empty chair in between? The answer seems obvious; farther is usually better. But suppose that, amid rows of empty seats, a stranger sits six feet away in the row in front of yours, but directly opposite and facing you. In this case, you might try to avoid looking up and accidentally meeting the stranger’s gaze. Even if you manage to avoid that, you are still an easy target for the stranger’s scrutiny. With this arrangement, the farther stranger is more intrusive than the closer one sitting two seats adjacent. Examples such as this are buttressed by research showing that physical spacing is just one factor among many in determining how people manage their involvement with surrounding others (Schaeffer & Patterson, 1980).

The advent of digital technologies has complicated how we navigate concepts such as closeness and involvement. In an earlier time, nonverbal behavior researchers would have been vexed to understand the intimacy of two letter-writers, a literal world apart, who exchanged the deepest of secrets over their lifetimes yet never shared a spoken word or gazed at each other’s countenance. Today, the vexation is compounded. What do we make of two people, sitting opposite one another, who are each video-chatting with others thousands of miles away? Who is closest to whom? McArthur (2016) noted the problems posed by *digital proxemics*, among them locating people and studying their interactions when they are partially or completely virtual.

### Misconception No. 2: Conclusion

Like body language, the construct of a stable, insulating personal space is a simplistic one that impedes our understanding of NVC and its everyday application. There is no doubt that we have personal boundaries and feel intruded upon when they are violated, but contrary to the popular personal-space account, those boundaries are not stable across partners and situations. Rather, preferred distances are malleable across the circumstances of interaction. Furthermore, distance is just one component in a system of behaviors regulating our involvement with others. Gaze, touch, facial displays, body orientation, lean, and posture combine with distance to do the regulatory work and signal a likely course of action (Crivelli & Fridlund, 2018; Fridlund, 2017; Patterson, 1983, 2011). This process is typically dynamic and interpersonal, not static and attached to specific people across encounters. Numerous factors precipitate these behavioral adjustments in the service of momentary conscious and unconscious goals. These factors include setting features, expectancies, personality (and other temperamental predispositions, culture, gender, and external incentives (Patterson, 2019).

## Misconception No. 3: We Have Basic Emotions That Are “Read Out” by Universal, Evolved, Iconic, Categorical Facial Expressions

The face is a picture of the mind with the eyes as its interpreter.

—Cicero

Was Cicero right to claim that seeing others’ faces was to know their minds? Can we see people’s faces and know what they really feel? Face reading to divine another’s inner life has a long history. Using faces to assess personality goes back at least to ancient Greece, but the idea that people’s faces mirror their real emotions was popularized by Charles LeBrun, court painter to Louis XIV (Montagu, 1994). LeBrun linked each of Descartes’s categorical “passions” to its own face, declaring that the face displays the passions as a clock does the time. He prescribed precisely how each face should be drawn, with detailed paintings and schematic drawings (Fridlund, 1994, 2021), under the presumption that his art fit all humanity and not just the French upper class.

### The doctrine of evolved universality

LeBrun’s legacy of stylized faces and their readout of matching emotions became received wisdom, and it lives on in today’s *basic emotions theories* (BET). These theories took hold in the late 1960s, mostly via Paul Ekman and his mentor Silvan Tomkins. Drawing upon Charles Darwin’s theorizing in *The Expression of the Emotions in Man and Animals* (Darwin, 1872), Tomkins and Ekman proposed six universal, evolved, categorical, eruptile emotions, with the list of these basic emotions has grown over the years (Ba˛k, 2016; Colombetti, 2014; Ekman & Cordaro, 2011; see commentary by Leys, 2017). Each emotion came with its own iconic face (e.g., smiles for happiness, scowls for anger, and pouty faces for sadness).

The major evidence used to claim the existence of evolved, universal basic emotions and their corresponding expressions came from a series of studies in which people were asked to select the best match of emotion terms or emotion-related stories to equal numbers of stagey posed faces, faces that eerily resembled LeBrun’s stylized portrayals. These were static faces, not the dynamic ones seen in everyday life. This was a method pioneered by Darwin (Snyder et al., 2010). Initially done with American participants (Allport, 1924; Tomkins & McCarter, 1964), these studies were extended to non-Western indigenous peoples, such as the Fore of Papua New Guinea (Ekman et al., 1969; Ekman & Friesen, 1971).

Ekman’s ready generalization of such findings led to this proclamation: “When someone feels an emotion and is not trying to disguise it, his or her face appears the same no matter who that person is or where he or she comes from” (Ekman, 1980, p. 7). Although respondents from a few Pacific indigenous societies showed overall agreement rates above chance levels, the studies and the conclusions based on them have been seriously contested (Crivelli & Gendron, 2017; Gendron et al., 2018; Nelson & Russell, 2013; Russell, 1994, 1995; cf. Ekman, 2017).

The idea that there was a set of faces reflecting the same emotions worldwide caught fire and was part of a 1960s Zeitgeist in which antiwar sentiment was brewing worldwide against the prevailing Cold War fever. It was in that emerging globalist context that those supposedly universal faces with their matching emotion terms found their way into nearly every introductory psychology text and were made into posters displayed on preschool walls (Crivelli & Fridlund, 2019). Cognitive-neuroscience researchers now use those categorical faces as probes to locate purported “emotion centers” in the brain (Celeghin et al., 2017; Morris et al., 1996; but see Lindquist et al., 2012). Artificial intelligence (AI) researchers use databases composed of such poses as training sets for AI applications intended to discern people’s emotions (Haamer et al., 2018; McDuff et al., 2016).

It now appears that the universality bandwagon was premature, and its endorsement of the Western received wisdom that certain categorical faces portray inner emotions was ill-founded on many grounds (Barrett et al., 2019; Leys, 2017; Ortony, 2021). The difficulties were apparent but underplayed in the original studies. In line with their authors’ preconceptions, cultural matching rates as low as 40% to 50% were considered accurate recognition of the presumptively termed “facial expressions of emotion” (Crivelli & Gendron, 2017; Russell, 1994, 2017).^8^ These low matching rates depended on the level of Westernization, and they were likely inflated by numerous technical factors (Nelson & Russell, 2013). More generally, the interpretation of those studies reflected the BET presumption that evolution promotes universality, whereas culture promotes diversity. This presumption is fallacious. Evolution readily creates diversity, a fact highlighted by Darwin himself in his *adaptive radiation*. Cultural transmission can easily account for any uniformity, not only through common learning, but also because we are all products of one long migration, and many cultural practices migrated with us (Crivelli & Fridlund, 2018, 2019; Fridlund, 1994; Richerson & Boyd, 2005).

### Acknowledging cultural diversity

Initially, BET’s foundational studies seemed to confirm a common set of facial prototypes expressing basic emotions, and these emotions and expressions were considered one of several human universals (Brown, 1991). Evolutionary psychologists used these studies to argue that basic emotions exemplified domain-specific psychological adaptations that evolved to solve everyday problems (Ekman, 1992; Tooby & Cosmides, 1990). Facial behaviors, used as proxies of basic emotions, were cast as outputs of categorical evolved psychological mechanisms (Shariff & Tracy, 2011).

In the 2010s, criticisms began to emerge regarding the generality of key findings, because of their narrow sampling of human cultures as well as their questionable assumption that Western lab-based experimental methods could be reappropriated for the study of diverse cultures (Crivelli, Jarillo, & Fridlund, 2016). This second-guessing of the early studies occurred in the context of a spate of failures to replicate highly publicized findings in the health, cognitive, and behavioral sciences. These failures resulted in a push to reform scientific practices to ensure the trustworthiness and reproducibility of science (Ioannidis, 2012; Open Science Collaboration, 2015).

Amid calls for reform, “expressions of basic emotions” remained on the shrinking list of human universals (Henrich et al., 2010), but not for long. Two independent multidisciplinary research teams ventured out to test the universality of the so-called facial expressions of emotion in four small-scale societies: the Trobrianders of Papua New Guinea, the Himba of Namibia, the Hadza of Tanzania, and the Mwani of Mozambique (Crivelli, Jarillo et al., 2016; Crivelli et al., 2017; Crivelli, Russell, et al., 2016; Gendron et al., 2014, 2020).

Findings from these studies revealed considerable cultural diversity in the faces people use and how they interpret and react to them. In addition, these studies used both classic forced-choice methods and innovative ones that allowed for more data-driven results using less constrained methods of inquiry (Gendron et al., 2015, 2018). The investigators were also more attuned to ethnographic issues sidestepped in the early BET studies but now mandated for internal and external validity, and they made sure to include diverse methods, samples, and collaborators (Medin, 2017; Medin et al., 2017). These studies in small-scale societies included extensive prior fieldwork to attain knowledge bases of cultural practices and conceptions (Kagan, 2007; Rai & Fiske, 2010), personal facility with the local languages, and hypothesis testing aligned with the in-field findings (Crivelli, Jarillo, & Fridlund, 2016).

Did these new findings overturn the vaunted universality of the “Ekman faces” and their presumed relations to emotion? Ekman (2017, p. 42) himself provided a new criterion to falsify the existence of a set of universal facial expressions of emotion: if “the expressions that the majority of people in one country judged as showing one emotion (let us say anger) were judged as showing another emotion (fear) by the majority in another culture. This never happened.” It did, but it took researchers outside the BET tradition to show it. Trobrianders of Papua New Guinea understood BET’s supposedly universal “fear” face as an agonistic threat display (Crivelli, Russell, et al., 2016), and that usage occurs not just among one exotic group of people but also in several African, Amazonian, and Pacific small-scale, indigenous societies (Eibl-Eibesfeldt, 1989).

### Do faces really express emotion?

The discovery of cultural diversity in how we use our faces has also led to a rethinking of the general idea that our faces are readouts of emotion. It turns out that the presumed concordances between faces and emotions, which BET had assumed were universal, may not exist even within industrialized societies (Jack et al., 2012). Moreover, a recent meta-analysis of studies of both coded BET-categorized facial expressions and emotion measured by its commonest proxy, self-report, showed that concordance between the two was modest to low (Durán et al., 2017; Durán & Fernández-Dols, 2021). Evidence from studies of facial behavior of infants and the congenitally blind, as well as research on both the production and perception of adult facial behavior, suggests that our faces resist categorical interpretation as indications of emotion (Barrett et al., 2019). How is this weak concordance possible when such a connection seems intuitive, at least to Westerners? Three factors undoubtedly contribute.

First, many faces taken as expressions of emotion are actually paralinguistic forms of social judgment and appraisal (Bavelas, 2007; Fridlund, 1994). This happens, for example, when “angry” faces relay condemnation and “sad” faces disappointment; “happy” faces condone, appease, and approve; “fear” faces signal submission; and “disgust” faces show revulsion. These faces act as running commentaries on, and sometimes interjections about, the actions or utterances of interactants. They are often quite independent of one’s “inner emotion” because their referents are external. So when we ask a friend, “How was the movie?” it is unlikely that her smiles or frowns are about some inner state; they are about the movie (cf. Fridlund, 1994).

Second, we seldom test our assumptions about what others’ faces indicate, nor do we have any ground truth by which to do so. We default to our culture’s received wisdom and turn our social beliefs into self-fulfilling prophecies (Merton, 1948). Only when we venture past those defaults do we discover that the smiler was humiliated, the scowler was physically injured, and the tearful person just received news that her cancer was in remission.

Third, we are wretched witnesses and historians regarding how we use our own faces. Indeed, all the problems of eyewitness testimony (e.g., people closest to a crime are often the least reliable reporters) are redoubled when people act as their own witnesses (Fridlund & Russell, in press). One clever study demonstrated just this point. Schützwohl and Reisenzein (2012) arranged for participants to arrive at their laboratory via a stark corridor. After some in-lab activities, they were asked to leave by the same door. When the participants left the lab, however, they did not see the corridor—they entered a small room painted bright green with a red office chair, a quick change of scene accomplished with movable prefabricated elements. Almost all participants reported being quite surprised and showing it on their faces, but the BET-stipulated “surprise” face was displayed by only 5% of them.

### What do faces really do?

If “facial expressions of emotion” really aren’t, then what are these facial movements? A clue came from dramatic developments in how biologists regarded the signals issued by nonhumans. During the 1950s and 1960s, ethologists such as Niko Tinbergen and Konrad Lorenz regarded animal communicative displays as the outputs of content-insensitive tripwire mechanisms triggered by releasing stimuli (Lorenz, 1967; Tinbergen, 1953). For example, red bellies on male stickleback fish provoked territorial aggression by other males, and displaced graylag goose eggs—or even golf balls planted by wily experimenters—triggered egg retrieval. In the 1970s, however, a new generation of ethologists found that these displays were not fixed or cartoonish eruptions but flexible, social, and contextual signals by which animals negotiated social encounters (Marler et al., 1986a, 1986b; Smith, 1977). On this basis, zoologist Robert Hinde (1985) questioned whether Darwin’s *The Expression of the Emotions in Man and Animals* (1872) was mistitled.

A new breed of *behavioral ecologists* rejected the view that animal signals were reflexive, or eruptions of categorical emotions, because no animal could survive for long if it kept issuing signals to its own detriment (Krebs & Davies, 1978; Maynard Smith, 1982). Rather, displays were understood as serving the interests of signalers within their social environments. Signaler and observer, even when they were predator and prey, became coevolved dyads in which displayers indicated their contingent behavior, and observers used display behaviors to predict the issuers’ next moves (Krebs & Davies, 1987; Krebs & Dawkins, 1984).

Beginning in the early 1990s, Fridlund (1991a, 1994) saw that behavioral ecologists were finding animal displays much more strategic and context-dependent than BET’s approaches were granting for human faces. He developed the *behavioral ecology view* of human facial expression (BECV) based on contemporary evolutionary principles. There are four main tenets of BECV.

First, it reconceives human facial displays, like those of animals, as indications of contingent intent rather than expressions of emotion. In other words, faces are social tools by which people influence their social interactants.^9^ Table 1 contrasts this functional view with the usual basic-emotions descriptions. Thus, people may show the prototypical BET “angry” face regardless of whether or not the displayer is angry. The face may be disapproving, deterring, disciplinary, part of a power-play to subordinate, or simply a sign of constipation or acid reflux. Similarly, people make the BET “sad” face to recruit succor or affirmation, whether they’re injured (“Mommy, it hurts!”), relieved (“My partner didn’t have a heart attack after all”), or chagrined (“How could you do this to me?”).

**Table 1.** Two Approaches to Common Facial Behaviors: Expressions of Internal Emotions Versus Functional Social Tools

Facial behavior	BET (emotion expressed)	BECV (social-tool use)
Smiling	Happiness	Influence interactant to play or affiliate
Pouting	Sadness	Recruit interactant’s succor, protection, or affirmation
Scowling	Anger	Influence interactant to submit
Gasping	Fear	Deflect interactant’s attack via one’s own submission or incipient retreat
Nose scrunching	Disgust	Reject current interaction trajectory
Neutral	Suppressed emotion Poker face, or no emotion	Lead the interactant nowhere in interaction trajectory
Microexpressions or compound expressions	Leaked or blended emotion	Conflict between displayer’s interactional tactics

Note: Adapted from “Facial Displays Are Tools for Social Influence,” by C. Crivelli and A. J. Fridlund, 2018, *Trends in Cognitive Sciences*, *22*(5), p. 394. Copyright 2018 by Elsevier Ltd.

BET = basic emotions theory. BECV = behavioral ecology view (of facial displays). *Social-tool use* refers to possible usage of common facial behaviors, cast in terms of behavioral consequence. Actual display behaviors and usages in BECV are dependent on interactant identities, histories, and the social context.

Second, BECV understands solitary faces as implicitly social in various everyday situations—for example, when people call out for rescue, pray to God, nurse their houseplants, curse recalcitrant computers, imagine attentive others, and praise themselves for their cleverness or performance (see Crivelli & Fridlund, 2019; Fridlund, 1991b, 1994; and Fridlund & Duchaine, 1996; for discussions of implicit and animistic interactions).

Third, BECV recognizes that natural selection and cultural selection can each generate commonalities or divergences via numerous mechanisms, and any human facial behavior will always reflect both nature and culture (Lindquist et al., 2022). No universality can be presupposed, nor can commonalities be assigned a priori to nature with divergences left to culture.

Last, BECV considers the idea that faces “leak” to reveal breakthrough emotion to be unverified and probably unverifiable. Glimpses of incongruous facial behavior, such as so-called microexpressions, instead signify momentarily conflicting intentions (we discuss this further under the next misconception). Thus, parents disciplining their children for finding their way into the cookie jar may glare at them to press the point yet betray a flicker of a smile to approve their cleverness.

### Misconception No. 3: Conclusion

The common-sense view that categorical emotions were causally linked to certain iconic facial behaviors was rooted in Western philosophical and artistic traditions regarding the “passions.” The authors of BET’s foundational studies in Papua New Guinea perpetuated the Western narrative and regarded it as the self-evident product of human evolution. They made the existence of universal categorical basic emotions and corresponding eruptive overt behaviors (i.e., putative facial expressions of emotion) a fundamental part of human nature, such as bipedalism or stereoscopic vision, and the expressions were likened to other purported universals, such as color perception or analog numeracy (Ekman, 1992; Henrich et al., 2010; Tracy, 2014).

In some respects, aspiring to prove universality in facial expressions—an all-or-none proposition—was always a tall order, because so few cultures were ever studied (Nelson & Russell, 2013; Russell, 1994), and because behavioral traits tend to show much more variation than morphological ones. The most conspicuous example is handedness. Worldwide, the prevalence of right-handedness is roughly 90%, far above the cultural matching rates in any facial-expression study, yet no one has spoken of the universality of dexterity; sinistrality and ambidexterity are recognized, stable variations (Crivelli & Fridlund, 2018). It therefore came as no surprise when recent studies in four small-scale African and Pacific societies failed to replicate BET’s canonical studies. The results, found in small-scale societies, were based on tests of both BET predictions and alternative hypotheses.

It appears that, with regard to facial expressions, the doctrine of universality has failed empirical testing. In the last decade, the data gathered in small-scale societies have extended our knowledge on the extent of diversity, context dependency, and flexibility in the behaviors that human beings use to negotiate encounters with others. These cross-cultural findings, which countered the presumption that human emotions were universally expressed on faces, were anticipated by Darwin (e.g., adaptive radiation in the Galapagos) and the behavioral ecologists who emerged in the 1970s.

We suggest that emotion may not be the best way to understand what we do with our faces. In the 1990s, BECV redefined how we conceptualize human facial displays using an externalist and functional perspective in a way that reconciles psychology with evolutionary biology, and it accorded humans the same subtlety and interactivity in their displays as modern theorists give to nonhumans.^10^

The fact that expression does not imply categorical emotion is brought home in human-computer interactions in which people interact with avatars, or simulated humans, in real or virtual worlds. Suppose a child is interacting with a pedagogical avatar as part of computerized instruction (Lin et al., 2020). If the avatar smiles at the child’s performance, do we conclude that the computer creating it is internally happy? And if the avatar scowls when the child uses blacklisted curse words, does its scowl mean that the computer is angry? Clearly, the avatar’s faces are intended to guide the child’s conduct. We believe that people’s faces work the same way.

This discussion does not and should not imply that cross-cultural research on facial expressions has become any less important. Commonalities and differences may emerge with detailed studies that do not favor either. Future studies of facial expressions should examine which faces occur, by whom, in what settings, in which societies. Such studies should proceed without undue theoretical burden, such as the stipulation that they express categorical emotions. In accordance with BECV and the systems approach we outline below, we believe that these studies should focus on how faces integrate with language and other nonverbal behaviors to regulate social interactions.

## Misconception No. 4: The Body Never Lies

The final misconception concerns the claim that distinctive, identifiable nonverbal behaviors are reliable indicators of deception. The role of nonverbal cues in detecting deception has long been a popular topic for researchers and the lay public. The phrase “the body never lies” reflects an implicit, and sometimes explicit, assumption that deception can be detected by some disconnect between the content of a lie and the speaker’s nonverbal behavior (Nierenberg et al., 2010). Where on the body those lies are supposedly detected has ranged literally from head to toe—from head movements and facial twitches to postural shifts and foot jiggling.

The notion has permeated Western popular culture, basic science, and high-stakes arenas such as global terrorism and counterintelligence, and it has become so longstanding and ingrained that streams of private and public funding now sustain a multibillion-dollar industry predicated on claims that liars can be caught by analyzing certain nonverbal behaviors. An August 2020 Amazon.com search for books on body language and deception turned up 305 results, including titles such as *Spy the Lie: Former CIA Officers Teach You How to Detect Deception* and *Detect Deceit: How to Become a Human Lie Detector in Under 60 Minutes* (https://www.amazon.com/s?k=%22body+language%22+and+deception&ref=nb_sb_noss_2).

Just as YouTube influencers tout what they call “body language” as key to success, they also guide their fanbases to learn how to spot deceit in in their partners, bosses, associates, and children. One YouTube channel, “The Behavior Panel” (https://www.youtub.com/channel/UCx_8ri2rYergbu_06VNSPlw), features the “world’s leading behavior experts” decoding videos of politicians and celebrities to divine what they really mean when they avert their eyes, twitch their lips or noses, sit straight or slump, and pause too little or too long when they speak. Is there any merit to such popular practices? To simplify our overview of research on nonverbal behavior and deception, we focus first on bodily movements and then on facial displays.

### Bodily movements and deception

Sigmund Freud often noticed that his psychoanalytic patients made off-task movements as they free associated or related their dreams. With a seeming lack of awareness, they fiddled with their watch chains, removed and replaced their wedding rings, or jiggled their pocket change. Freud termed these “symptomatic and chance actions” *parapraxes*, and he considered them revelations of unconscious material that conflicted with what was conscious (Freud, 1901/1915). Some nonverbal behavior researchers used the same logic to claim that bodily movements divulge the truth while the mouth tells the lie. The belief that lies are transparent dates back nearly 3,000 years and sees currency in the legal system, where jurors are instructed to notice the nonverbal behavior of people in the witness chair (Vrij et al., 2019).

Could Freud’s conflict formulation, minus its conscious/unconscious corollary, explain the bodily movements held to indicate deception? Ethologists have long observed that animals show “conflict behaviors” when they are at behavioral junctures. To deter interlopers, birds at a territorial boundary must choose either to charge across the boundary or stand their own ground inside it, and they often displace or redirect the conflict by preening, pecking the ground, or plucking the grass (Alcock, 1984, Fridlund, 1994). Numerous studies have shown that increased psychological stress results in greater body muscular activity. Temperamentally anxious people tend to be tenser and more agitated as well (Fridlund et al., 1986; Hazlett et al., 1994; Jung et al., 2016). Might this stress account for the supposedly telltale bodily signs of deception? Trivers (2011) suggested three reasons, all related to stress, why there might be such signs: (a) “because of the negative consequences of being detected . . . people are expected to be nervous when lying”; (b) because concern over appearing nervous may lead people to “exert control, trying to suppress behavior” leading to “overacting, overcontrol, a planned and rehearsed impression, or displacement activities”; and (c) because the cognitive requirements or “load” of lying means that liars “think too hard,” which has behavioral repercussions (p. 10).

How exactly would those factors be evident? Here we find a vast amount of lore regarding bodily signs of deception. Trivers suggested that deception would be accompanied by less blinking, fidgeting, and hand gesturing, but longer speech pauses and increased vocal pitch (Trivers, 2011, pp. 10–12). In *Spy the Lie: Former CIA Officers Teach You How to Detect Deception* (Houston et al., 2012), the authors descried the “behavioral myths” that pervade the field (p. 25) yet contended that being inappropriately polite (p. 38) is a clue, as is gesturing that hides the mouth or eyes. Throat clearing or swallowing is another giveaway, as are biting or licking the lips, grooming actions like hand-combing the hair, and “sweat management” such as hand-wiping the brow or pulling out a handkerchief to do it.

Were Trivers’s suppositions on target? Are the CIA retirees in *Spy the Lie* telling the truth? Unless various intelligence services have conducted top-secret validation studies,^11^ we must be content with publicly available research, and it paints a starkly different picture. The consensus of deception researchers is the one reached by Charles Bond and Bella DePaulo in their analysis of over 200 studies of judgment accuracy in nonverbal detection of deception. This analysis found that judges were no more accurate “than would be expected by chance, and the best judges are no more accurate than a stochastic mechanism would produce” (Bond & DePaulo, 2008, p. 477).

Most of these studies had observers make global judgments about deceit and did not explore what specific behaviors informed their judgments. Isolating those behaviors was the goal of a massive earlier meta-analysis by DePaulo et al. (2003), who compiled 120 separate data sets from 116 studies that encompassed nearly a dozen ethnic groups and found roughly 100 behaviors that were predominantly nonverbal. Restricting these cues to ones that emerged in more than six studies, 50 behaviors remained. Effect sizes were computed on the basis of mean occurrences of those behaviors in deceptive versus nondeceptive conditions. Only 14 of the 50 cues were statistically significant discriminators of potential detection, with the standout cue being “verbal and vocal immediacy . . . [the] degree to which responses sound direct, relevant, clear, and personal.” Following that was “discrepancy or ambivalence” in verbal and nonverbal presentation.

In their summary of DePaulo et al.’s (2003) most relevant findings, Vrij et al. (2019) observed that most cues that were at least partly nonverbal showed no relationship to deception whatsoever. For the ones that did, the effect sizes were small, leading Vrij et al. to the dismal conclusion “that those cues are mostly unrelated or, at best, weakly related to deception” (p. 302). Even this weak relationship is suspect. Most detection-of-deception contexts are likely to engender stress in both the innocent and the guilty, and it is crucial to remember, consistent with Trivers’s (2011) cautions, that any indications of stress can be interpreted in multiple ways. People may be stressed not because they are lying but because they fear being accused of it (rightly or wrongly), resent the fact that they are suspected of it, or are simply fraught at being put on the spot about it.^12^

So what do we make of the evidence? Overall, it appears that “liars” give off nonverbal behaviors while they are lying. But such nonverbal behaviors do not certify their lying, because both liars and nonliars may give off the same nonverbal behaviors for reasons other than lying (i.e., when they are anxious). Given this state of affairs, Vrij et al. (2019) noted the patent, persistent, disturbing discordance between such findings and the current practices of so-called lie-detection experts: “Lively debates about the merits of nonverbal lie detection no longer take place at the scientific conferences that we attend. Yet nonverbal lie detection remains highly popular among practitioners, such as police detectives, and in the media” (p. 302). As we shall see, this same disconnect between the evidence on bodily movement and deception and its unwarranted application extends to facial displays and facial deception.

#### Bodily deception and pseudoscience

In light of the overwhelming evidence debunking the misconception that the “body never lies,” it may be unsurprising that commonly used detection-of-deception programs based on the misconception do not fare well. An important critical review captured the prevalence of this misconception and pulled no punches in slamming much of nonverbal-behavior detection of deception as unalloyed pseudoscience (Denault et al., 2020). Among the egregious offenders was the most common behavior-oriented protocol, the Behavior Analysis Interview (BAI). The BAI involves a nonaccusatory interview at first, followed by an Inquisition-like confrontation consisting of 15 standard questions intended “to elicit an initial admission of guilt” (Inbau et al., 2013, p. 294, cited by Denault et al., 2020, p. 4).

Certain examinee nonverbal behaviors in the BAI interrogation are stipulated to be signs of deceit, including maintaining a closed, withdrawn posture, sitting askew in the chair, and leaning forward constantly. Opposite movements and postures indicate honesty. Lack of eye contact and gaze aversion are likely to indicate the withholding of information, a clear departure from numerous findings indicating no relationship to deception. These behaviors are judged to indicate guilt on the basis of the BAI’s declaration that guilty parties will be more stressed by interrogation than innocent ones. This assumption may hold in some cases but is clearly unfounded in many others. For example, recidivists judged guilty yet again may be far less stressed at the prospect than innocent people who are falsely judged guilty. For them, the consequences could be catastrophic. As evidence of the flimsiness of the BAI’s rationale and practice, the only empirical investigation of the BAI in which the ground truth of guilt or innocence was known—a mock theft analogue study—found that BAI results could not discriminate the guilty from the innocent (Vrij et al., 2006).

Perhaps no psychological theory has ever been tested at greater effort and expense—and gotten worse results—than the program for Screening of Passengers by Observational Techniques (SPOT) by the U.S. Transportation Security Administration (TSA). Introduced in 2006 and premised on the claim that “behavioral indicators . . . can be used to identify persons who may pose a risk to aviation security” (U.S. Government Accountability Office [GAO], 2013), the TSA deployed about 3,000 behavior detection officers across 176 U.S. airports. These officers were trained to observe airline passengers at prescreening using a 92-item checklist of criteria that included exaggerated yawning, mouth-covering, a bobbing Adam’s apple, excessive throat clearing, rapid blinking, complaining more than usual about the screening process, whistling while approaching screening, gazing down, a pale face in males from recent shaving, and the rubbing or wringing of hands (Winter & Currier, 2015).

Denault et al.’s (2020) review of pseudoscience in nonverbal behavior detailed SPOT’s ignominious failure on field testing. Similarly, the GAO’s summary judgment on SPOT concluded that “meta-analyses and other published research studies we reviewed do not support whether nonverbal behavioral indicators can be used to reliably identify deception” (GAO, 2013, p. 15). The outcome data from SPOT might have been anticipated given the paucity of evidence that nonverbal behaviors were reliable indicators of deception per se (Bond & DePaulo, 2008; DePaulo et al., 2003).

The GAO (2013), in its internal review of 400 separate studies related to detecting deception, noted that “the ability of human observers to accurately identify deceptive behavior based on behavioral cues or indicators is the same as or slightly better than chance (54 “percent”)” (p. 16). Moreover, the 178 sources of evidence the TSA used to justify its behavioral indicators boiled down to only three original research articles, and these few articles only supported the use of some of the indicators comprising the TSA’s checklist. The GAO’s overall assessment? “Decades of peer-reviewed, published research on the complexities associated with detecting deception through human observation called into question the scientific basis for TSA’s behavior detection activities” (GAO, 2013, p. 47). As Denault et al. (2020) indicated, SPOT cost U.S. taxpayers an estimated $1.5 billion for 2007 to 2015, with little to show for it. Did the TSA disband SPOT as a failed program? As with many government programs, ineffectiveness has not compromised longevity, and SPOT seems simply to have reemerged under the radar as a new TSA surveillance program called Quiet Skies.

### Facial deception: discordant displays and microexpressions

In detecting deception, does the face provide better clues than the body? The dominant theory of faces, BET, claimed that we deceive with our faces in two ways—by making faces discordant with how we feel, and by showing intrusive facial behavior that reveals emotions we try to suppress. We summarize and show fatal problems with both.

#### Discordant displays

The presumption of authentic face-emotion links in BET widened the scope of deceit to unprecedented phenomena. Under the BET position that individuals feeling a basic emotion and not trying to conceal their feelings produced the same facial expressions across societies (Ekman, 1980), BET researchers concluded that people whose emotional states did not match their facial expressions were lying about their feelings with their faces (Ekman & Friesen, 1975). From this perspective, bursting into tears at discovering one’s child was not critically ill became deceptive, because a teary-eyed face is supposed to signal inner sadness.

Among the different facial displays that were considered universal expressions of emotion, the study of smiling has been pivotal. Under BET assumptions, for example, smiling at work while in a cranky mood would be deceptive, because authentic or “felt” smiles arise only with happiness (Ekman & Friesen, 1982). However, this perspective does not take into account that the cranky person, though irritable, might also want to be authentically courteous; it turns everyday politeness into mendacity (Fridlund, 2017). BET studies of facial deception nonetheless began promoting the idea that smiles accompanied by wincing, the so-called Duchenne smiles, were authentic, felt, and spontaneous, whereas those smiles without wincing were “unfelt,” deliberate, and therefore false and phony (Ekman et al., 1990). This claim was accepted uncritically, despite the original report’s lack of discriminant validation and the fact that wincing in the Duchenne smile was an artifact of stimulus intensity and not hedonics (Fridlund, 1994).

Indeed, subsequent research has shown that, contrary to BET proclamations, Duchenne smiles (a) are at least as affected by sociality as non-Duchenne ones, and occur frequently in highly scripted social encounters; (b) can be produced easily on request; and (c) occur as a function of both smile intensity and stimulus intensity regardless of valence (Crivelli & Fridlund, 2019; Fernández-Dols & Carrera, 2010; Girard et al., 2021; Krumhuber & Kappas, 2022; Krumhuber & Manstead, 2009).

#### Microexpressions

*Micromomentary expressions* were first discussed by Haggard and Isaacs (1966), who reviewed videotapes of psychotherapy patients. They found flashes of facial behavior that interrupted more sustained expressions and were noticed mostly when the playback was slowed. Like Freud with his parapraxes, the authors saw these glimpses, which lasted only a fraction of a second, as revelations of suppressed content. Other researchers noted similar therapy-related behaviors and claimed that these fleeting facial movements revealed deception (Ekman & Friesen, 1969). Microexpressions, however, arose as a post hoc explanation for the results obtained in a well-known study with nurses (Ekman & Friesen, 1974a, 1974b). The paradigmatic study, reviewed by Fridlund (2021), had two experimental conditions. In the honest condition, female nursing students individually watched excerpts of a pleasant film with an interviewer present who asked participants to “truthfully describe their reactions” during the film. In the dishonest condition, the students were asked to watch a medical film detailing amputations and severe burns and to “conceal negative affect” during the film. In the dishonest condition, the questioning was confrontational (“What kinds of feelings are you having right now?”; “Are you telling me the truth?”; “Do you think I believe you?”; Ekman & Friesen, 1974a, p. 291). Untrained student observers who viewed video snippets could not distinguish honest from deceptive instances on the basis of facial behavior. One decade later, these researchers replicated the nursing studies, finding similar unimpressive results (O’Sullivan et al., 1988).

Rather than accepting these null findings, the researchers faulted their judges, claiming that these microexpressions were so brief, with durations from 1/25 s to 1/5 s, that their untrained observers would naturally have missed them. To prove their point, they commissioned “four experienced facial analysts,” all unnamed, to judge the nursing students’ recorded faces, and they reported that these experts accurately judged both the honest and deceptive behavior in most of the trials (Ekman & Friesen, 1974a). These findings came with no description of the procedures used, the judgment criteria, the specific outcome data, or any assurance that the scoring was blind. Needless to say, these claims were greeted with skepticism (see Bond, 2008; Bond & Uysal, 2007; cf. Ekman et al., 2008).

The idea that microexpressions are to be seen in human faces gained traction mainly on the strength of such anecdotal evidence, and found its way into basic and applied science and self-help trade books. Eventually introductory psychology, criminology, and forensic texts mentioned microexpressions, and the range of applications soon extended from national security to marital relations and personnel recruitment (Ekman, 2003, 2009; Gladwell, 2005; Li et al., 2018; Navarro & Karlins, 2008).

These developments transpired years before the first independent targeted investigations of microexpressions and deceit (Porter et al., 2012; Porter & ten Brinke, 2008; K. ten Brinke & Porter, 2012; L. ten Brinke et al., 2012). Porter, ten Brinke, and colleagues had participants view slides of various emotional-related stimuli while facing a camera that recorded their facial behavior, with instructions to “falsify,” “simulate,” or be “genuine.” Matsumoto and Hwang (2018) summarized these studies as showing (a) that microexpressions are quite rare, occurring in only 2% of all expressions (Porter & ten Brinke, 2008); (b) that the studies did not distinguish genuine from feigned remorse (L. ten Brinke et al., 2012); and (c) that the studies did not separate truthful from deceitful individuals regardless of stimulus intensity (Porter et al., 2012). The final result stood even when judges were shown internationally televised videos of people pleading for the return of missing relatives, with half the pleaders having actually murdered the relatives themselves (K. ten Brinke & Porter, 2012). Finally, controlled research on one well-established set of microexpression “training tools” found that, with training, overall accuracy at detecting deception was slightly below chance (Jordan et al., 2019).

#### Facial deception in context

As we noted earlier, all these studies of faces and deceit were bizarre distentions of the concept of deception, in that deviations from *theory-driven* predictions were made criterial. It was assumed that participants experienced certain emotions because of situations contrived to produce them (whether participants were exposed to face photos or videos or staged scenarios), and it was assumed that the experienced emotions would produce certain stipulated faces. Suppressed emotion, it was also stipulated, would leak onto the face, and so instructions to suppress, falsify, or neutralize the predicted faces to produce microexpressions were pitting purposeful actions against natural faces, with any incongruities reflecting the latter’s irrepressibility. This conflict between willfulness and authenticity was said to emanate from a neurological tug of war between competing brain structures (Matsumoto & Hwang, 2018; Matsumoto & Lee, 1993).

All this theorizing was unnecessarily complex and inattentive to the social demands of the experimental context. The nurses’ study (Ekman & Friesen, 1974a), like the many procedural variations used subsequently, was more prosaically understandable in terms of the instigation of ordinary conflicts in impression management. Simply put, nursing students were led to make faces that both reassured others (nurses must be empathic, and the participants were eager to become nurses) and showed stolidity (good nurses must conceal their discomfort from patients). If the “four experienced facial analysts” of the nurses’ study indeed observed microexpressions, then those signs were merely conflict behaviors arising from situationally contrived ambivalence, not telltale leakages of suppressed emotions (Fridlund, 2021).

### Misconception No. 4: Conclusion

Can we accurately “read” the nonverbal behavior and microexpressions of partners who have cheated, children who stole cookies from the cookie jar, or defendants who killed victims they insist they never met? Research evaluating the use of bodily movements to detect deception has turned up either null or minimal results. Literature reviews and meta-analyses show that facial microexpressions are infrequent, and inferences about them readily lead to both false negatives and false positives (Burgoon, 2018; DePaulo et al., 2003; Hartwig & Bond, 2011). Studies intended to be about deception per se often missed the mark precisely because they did not take into account the contextual factors that led to stress and ambivalence in their participants, signs of which were mislabeled “deception.”

The misguided belief that we can reliably detect deception using either the body or the face has been fueled in part by the lay conviction that people should be able to tell when they are being deceived, as the unpleasant truth leaves them vulnerable.^13^ Yet another reason this belief persists lies in the fact that there is money to be made by claiming that one can teach how to detect deceit, and there is a history of flawed science supporting that enterprise (Jupe & Denault, 2019; Jupe & Keatley, 2020). This creates a conflict of interest that jeopardizes the integrity of both research and its application (Chivers, 2019).

What will happen to the understanding of deception when we participate as our own digital avatars in the metaverse? As we interact with virtual others, will we continue to believe that we can see deception in the synthetic representations of others? Or will we “world-switch” here, too? Will we learn to base judgments of others’ truthfulness on evidence other than their electronically replicated nonverbal behavior, as we should have done long ago in the real world? Or will a virtual jurisprudence evolve by which nonverbal indicators like on-screen gauges, possibly superimposed on virtual others, signal their credibility and ours, with virtual penalties for computer-detected instances of virtual deception?

## Nonverbal Behavior Without the Misconceptions: A Systems Approach

In this article, we have discussed four common misconceptions about NVC. In our view, it is time to move beyond several ill-founded beliefs: (a) that NVC is a language; (b) that individuals possess a stable personal space that regulates their in-person contacts with others; (c) that our emotions are read out by universal, iconic, categorical facial displays; and (d) that the body never lies. From our vantage point, the Internet and social media have perpetuated these misconceptions, making claims that go well past the evidence. Propelled by obvious incentives, some professionals have used dubious science to promote practices that are unfounded, unreliable, and expensive.

### Countering the misconceptions

How might we clear the field of these misconceptions, provide a better framework for research, and accurately represent our results to the public? The replication crisis in psychology and other sciences has led to increased skepticism about high-profile findings with large payouts but dubious evidential bases. As we have seen, well-known meta-analyses on detection of deception were largely ignored, and it took the failure of a $1.5 billion U.S. government program to bring the cautionary research to public attention. Our most specific recommendation is that such high-profile endeavors should receive the earliest and most thorough scrutiny. Of course, this is no guarantee that the field of NVC will be purged of either bad science or frank pseudoscience.

More generally, what we propose is not an alternative set of dogmas, but rather a systems approach to research and theory that stimulates wide-ranging inquiry. An example of this kind of approach is a recent model of dyadic nonverbal interaction (Patterson, 2019). In general, the systems model describes and explains the dynamic interplay among individual, dyadic, and environmental processes in nonverbal interactions. That is, any particular outcome, whether it is a nonverbal display, a judgment of others’ nonverbal behavior, or a combination of both, is best understood as emanating from a network of interrelated processes. Although the details are beyond the scope of this article, the systems model embraces three principles that undercut the misconceptions we have described. Specifically, the model emphasizes that NVC engages multiple cues and behaviors concurrently; that NVC is interactive; and that context is critical, with the physical setting staging all our interactions, and with culture always the deep context. We review the importance of all three ideas.

### Multiple cues and behaviors

NVC is the product of multiple cues and behaviors (Patterson, 1995, 2011). On the sending side, individuals at any given point in an interaction display a variety of appearance cues and initiate a complex of behaviors. On the receiving side, individuals have a complementary role, perceiving a wide range of others’ appearance cues and behaviors. Of course, not all available cues and behaviors may register, and some of them may be weighted more heavily than others (Patterson, 2019). Simultaneous sending and receiving of such cue-behavior patterns occur even in brief interactions. To assume that any one behavior in isolation (e.g., a nose touch, or altered gaze) is part of a body language with invariant meanings misrepresents the configural nature of the multiple components that comprise NVC. Thus, the meaning of a specific action or display depends on the overall cue-behavior pattern. Likewise, the misconception of personal space results from an inattention to the multiple components (e.g., gaze, body orientation, facial displays, or other related behaviors) that determine the meaning and impact of NVC.

### Nonverbal communication is interactive

Our focus here is, of course, in social settings, but we must reexamine the boundaries of what is “social.” We cannot overlook the implicit sociality of the verbal and nonverbal behaviors that occur when we are physically alone, such as cursing at flaky computers, praying to God, rehearsing talks, plotting revenge, and pampering houseplants (Crivelli & Fridlund, 2018; Fridlund, 1994; Fridlund & Duchaine, 1996). That people can be physically alone but essentially social was always true for letter writers, even though the communication was lagged. People who are passive viewers of others’ nonverbal interactions, whether in public or on social media or TV, assume the role of bystanders, and the interactants’ knowledge that there are bystanders (i.e., audiences) will affect their behavior.

In standard in-person social settings, however, NVC is a two-way street with interactants reciprocating appearance cues and a stream of nonverbal behavior. Such reciprocation does not require sustained interaction. It can happen in very brief encounters in which people simply share the same setting for just a few seconds. These are the unfocused interactions we reviewed previously. Even in these incidental interactions, the nonverbal behaviors are complex and open to multiple interpretations. A smile toward the boss in the office hallway may be meant as ingratiation, whereas that same smile may be flirtation toward a co-worker. Nor are the impacts of such behavior invariant. To the boss, the smile may be seen as ingratiation, friendliness, appeasement, or subversion. For the co-worker, it may be taken as friendliness, healthy interest, or sexual harassment.

Whether interactions are focused or unfocused in nature, the systems model views nonverbal interactions as goal-oriented behavioral exchanges shaped by interdependent perceptual, cognitive, and affective processes between partners (Patterson, 2019). A failure to achieve goals increases the probability of behavioral adjustments or an early termination of the interaction (Patterson, 2019). Thus, the systems model provides an interactive perspective on NVC that stands in stark contrast to the misconceptions discussed in this article.

### The criticality of context

The four misconceptions we describe generally ignore the fact that all patterns of NVC are situated within specific interaction contexts. Two aspects of such contexts, physical environment and culture, deserve far more attention.

#### The physical environment sets the stage for interaction

We have previously detailed the role of the physical environment in our treatment of the misconception of personal space. The influence of the physical environment on social interaction is much broader, however. With some important exceptions (e.g., Altman, 1975; Barker, 1968; Oishi & Graham, 2010; Wicker, 1979), it has been sorely neglected in psychology generally, and in research and theory on NVC specifically (Patterson & Quadflieg, 2016).

The physical environment shapes NVC in complex and sometimes subtle ways. The dynamics of behavior settings, a central construct in ecological psychology, illustrate these influences (Wicker, 1979). Behavior settings are bounded geographical areas in which components such as the physical environment and behavioral norms collectively serve to facilitate ordered trajectories of events and behaviors over a limited period of time (Wicker, 1979). In such settings, whether they are college classes, office meetings, political rallies, or religious services, most people behave in line with the physical and social constraints of the immediate environment rather than acting in ways that dramatize their personalities, attitudes, or motivations. Individuals migrating across settings change their behavior, both verbal and nonverbal, to suit the constraints and expectancies of the new settings. Furthermore, because people select settings and settings often select people, individuals who stray too far from the setting norms may be unwelcome (Wicker, 1979).

Next, specific features of the physical environment also influence the give-and-take of NVC (Patterson & Quadflieg, 2016). For example, the design and arrangement of furniture in a setting delimit the options for interpersonal distance and orientation in seated interactions. In turn, distance and orientation affect impressions and ease of communication within a group (Altman, 1975; Patterson, 2021).

The measurable effects of subtle environmental features on NVC are discussed at length elsewhere (Patterson & Quadflieg, 2016), but a few examples suffice. Dimmer ambient lighting decreases how much detail we see in others’ appearances, and this lack of distinctiveness may increase the probability of “they are all alike” stereotyping (Cloutier & Macrae, 2007). Transparent glass barriers designed to separate or isolate people provide visual spaciousness but can decrease privacy (Marquardt et al., 2015). Pleasant citrus scents can facilitate trust and reciprocity between strangers (Liljenquist et al., 2010). Loud ambient noise is likely to drive people closer together so they can hear each other speak (Lloyd et al., 2009). NVC can occur in absentia, because people who have left the scene leave physical traces and objects (e.g., the magazines they opened to read, or the food they failed to discard) that are informative about their attitudes and interests to those who remain (Gosling et al., 2008; Webb et al., 1966).

Taken together, all these physical features shape the social interactions that occur amid them. Thus, the extent of this influence undercuts any mythical notion that nonverbal behaviors have invariant meanings across settings. A systems model will be required to understand current and upcoming video and metaverse modes of communication, which retain many of the features of in-person interaction but situate it in novel and sometimes otherworldly virtual settings.

#### Culture is the deep context

Just as human cultures have evolved their own languages, so too have they evolved their own systems of nonverbal displays. The diversity of modes of NVC in various cultures was a persistent theme in Darwin’s *Expression* (Darwin, 1872). Furthermore, anthropologists have documented exquisite diversity in the social roles, traditions, rituals, and social behaviors of indigenous peoples worldwide. For instance, among Australian Aboriginals, some groups use body-painting to signify their bloodlines; others inflict scars to signify social status. Unlike Westerners, who generally prefer their conversations face-to-face, some indigenous groups (e.g., the Guugu Yimithirr of northern Queensland, the Tenejapan Mayans in southern Mexico), find this confrontational and prefer speaking side to side or front to back (Levinson, 2003). Several Amazonian indigenous groups in Bolivia point using lip protrusion rather than hand or head movements (Key, 1962; Reiter, 2014). As yet another example of human diversity, the Wolof of northwestern Senegal regulate taking turns in seated conversations in part by grabbing the feet of their interlocutors (Meyer, 2014).

Finally, we return to the BET presumption that there are universal emotions that we all experience in the same way, even if culture intercedes to modify the supposed universal faces expressing them. There is ample cause to question this assumption as well. If our language concepts bear any relation to our experience, then continuing to argue the case for universal emotions will be tough indeed.

What are the roles of biology and culture in shaping nonverbal behaviors? In making culture only a thin veneer over a fundamental, overriding biology, BET drastically oversimplified the respective roles of both (Lindquist et al., 2022). Certainly, there are examples that fit BET’s universalist mold. People the world over have propositional speech, bipedal gaits, and opposable thumbs, and they yawn when bored or tired. These commonalities are all part of our biological heritage. But people also show great diversity in their music, cuisine, and clothing, and these are all aspects of enculturation.

In equating universality with biology and diversity with culture, BET ignored the ready examples that ran counter to its presumptions. For example, people show great diversity in their blood hemoglobin types, proportions of fast versus slow striate muscle fibers, and types of earlobes, and this diversity is also part of our biological heritage. Yet all peoples have weddings, use money, and cook with fire, and these universals are distinctly products of culture. Such commonalities would be expected, because humanity seems to have been the product of one long migration in which useful cultural practices tagged along. Geographic and other barriers can result in relative cultural and reproductive isolation, however, and so different cultures, in accommodating to changed circumstances, can diverge both in their genotypes and their practices.

Indeed, natural selection and cultural selection are now recognized as ongoing intertwined processes. Commonalities or diversity can result from either. Assignments to either biology or culture are likely to be oversimplified, as the kinds of analyses required to make those assignments—molecular genetic analyses of cultural phenotypes—are complex and do not admit of easy answers themselves (Fridlund & Russell, in press). The upshot is that when we examine how different cultural groups communicate nonverbally, we should not presume either commonality or diversity; we should be equally prepared to find either. Cross-cultural research, we suggest, should proceed in such a data-driven manner, without Western theory-driven preconceptions about likely findings. This will lead to a deeper and more comprehensive understanding of how diverse cultures communicate nonverbally.

## Conclusion

We have reviewed four common misconceptions about NVC—that people (a) communicate using body language; (b) have a stable personal space; (c) use universal, evolved, iconic, categorical facial displays to express underlying categorical emotions; and (d) give off, and can detect, reliable telltale clues of deception.

We are not making an indictment of the field of NVC, which has made great strides based on good science. Rather, we present a focused critique of certain presumptions related to NVC that persist despite weak evidential bases and remain pernicious influences on professional practices, research conduct, and lay understandings of the field.

To counter these misconceptions and help prevent new ones, we propose a systems approach to NVC that centers on the interrelations of nonverbal cues and behaviors, rather than their roles in isolation; emphasizes that communication is fundamentally interactive, not unilateral; and acknowledges that the context of communication must include the form of the immediate physical environment and the interactants’ cultural frames of reference.

## Transparency

*Action Editor:* Tina M. Lowrey

*Editor:* Klaus Fiedler

*Author Contributions*

M. L. Patterson, A. J. Fridlund, and C. Crivelli conceived, prepared, reviewed, and edited the manuscript. All authors approved the final manuscript for submission.

## Notes

1. These three features are most relevant to our exposition, but they do not exhaust other aspects that linguists find in formal languages, such as arbitrary relationships between words and referents (onomatopoeia being a prominent exception), combinatoriality (the ability to make new words from existing ones), and precision translatability, both among languages and in the transformation of expression from speech to writing and signing (e.g., Bouchard, 2013).

2. Gestures such as the “OK” sign, the extended third finger, the tongue inserted in the cheek, and the exaggerated nose scrunch act as iconic substitutes for speech. They function as part of language (Goldin-Meadow & Alibali, 2013), with more than 90% occurring in the presence of speech and facilitating speakers’ production of speech and listeners’ comprehension of its content (Cartmill & Goldin-Meadow, 2016; Krauss, 1988). Thus, given their linguistic nature, one that includes many aspects of formal languages (McNeill, 1985), such gestures might uniquely qualify as body language, but they are not among the nonverbal behaviors that usually comprise NVC.

3. Even here, some animals are 100% dog but have three legs (they are “tripawed”), and African Basenjis do not bark. Other (English) exceptions to 1:1 mappings of words onto meanings include homonyms in vocal speech (like *rain*, *rein*, and *reign*) and polysemic words in speech and writing (like *pen* and *mean*). *Dog* itself is polysemic, as one can “dog” (or hound) another by relentlessly following him, and doing so makes one a “dog” (“You ain’t nothin’ but a hound dog”). Polysemy is usually rapidly disambiguated by a word’s context. Thus, the meaning of “Do you have a pen?” differs depending on whether the query is followed by “I need to sign my check” versus “I need a place to put my cattle.”

4. Discussion of the relative left-hemisphere predominance in speech should not minimize the right hemisphere’s parallel involvement. Recent data suggest that the left hemisphere may govern speech timing and sound transitions (e.g., consonants to vowels), with right-hemisphere mediation of spectral aspects of speech, such as intonation and prosody (Floegel et al., 2020).

5. Searches were conducted on June 15, 2021 (https://www.proquest.com), updating earlier findings by Sommer (2002).

6. This unprecedented stable interpersonal boundary took the form of the “6-foot rule” mandated by the U.S. Centers for Disease Control to prevent the spread of COVID-19 in indoor spaces, along with the “1-meter rule” adopted by the World Health Organization and the “2-meter rule” adopted in the United Kingdom. These fixed boundaries were ultimately found insufficient to stop the spread of COVID (Bazant & Bush, 2021).

7. Exceptions occur amid longstanding antagonisms among family or disparate social-group members. Spatial arrangements dependent on group identities also occur in caste or monarchic systems, in the self-segregation of racial and ethnic groups, among different age groups of students, in seating and section classes of theater-goers or airline and cruise passengers, and with people or groups that require protection.

8. Ekman and colleagues explained the departures from universality by claiming that the training and traditions within various cultures resulted in on-the-fly modifications of the supposedly natural and biological faces (Ekman et al., 1969; Ekman & Friesen, 1969). Such *display rules*, an idea that originated with Wundt (1894), were invoked ad hoc to explain low matching rates but never the high ones, and the experimental procedures and evidence used to support the operation of display rules were incompletely reported and fatally flawed (Fridlund, 1994; Leys, 2017).

9. Scarantino et al. (2021) cast facial displays as appeals, but by signaling probable action the displays likely function more as nudges.

10. Since the introduction of BECV (Fridlund, 1991b, 1994), numerous theories have been proposed to counter or modify BET by ratifying BECV’s focus on our facial displays as functional and interactional. Recent examples of these approaches are Elfenbein’s *dialect theory* (Elfenbein et al., 2007), Keltner and Oatley’s (2022) *social functional theory*, Niedenthal’s *social-functional framework* (Martin et al., 2017), Scarantino’s (2017) *theory of affective pragmatics* (Scarantino, 2017), and Scarantino et al.’s (2021) *appeal theory*.

11. This is not farfetched, especially as agents of intelligence services such as the U. S. Central Intelligence Agency use interrogation techniques that are otherwise unavailable to the public. We also note that intelligence services have an interest in claiming they can spot deception just to intimidate guilty suspects into confessing. From this admittedly dark vantage point, the negative findings on detecting deception, though accurate, may give comfort to the guilty.

12. This lack of discriminant validity undercuts recent efforts to apply computer vision and machine learning to identify deception nonverbally, using training sets composed of videotapes of people known to be lying (e.g., Carissimi et al., 2018). Again, people may show certain nonverbal behaviors while lying, but not necessarily because they are lying, and the evidence suggests that the same behaviors occur outside of lying.

13. Fridlund (2021) contrasted our everyday assumption of transparency with the unpleasant fact that most people are by and large inscrutable, and he quoted Malcolm Gladwell in *Talking to Strangers* (2019, p. 162): “Transparency is a myth—an idea we’ve picked up from watching too much television and reading too many novels.”

## References

[bibr1-17456916221148142] Alcock J. (1984). Animal behavior (3rd ed.). Sinauer.

[bibr2-17456916221148142] Allport F. H. (1924). Social psychology. Houghton Mifflin.

[bibr3-17456916221148142] Altman I. (1975). The environment and social behavior. Brooks/Cole.

[bibr4-17456916221148142] Altman I. Chemers M. M. (1980). Culture and environment. Brooks/Cole.

[bibr5-17456916221148142] Ambady N. Rosenthal R. (1992). Thin slices of expressive behavior as predictors of interpersonal consequences: A meta-analysis. Psychological Bulletin, 111, 256–274. 10.1037/0033-2909.111.2.256

[bibr6-17456916221148142] Ba˛k H. K. (2016). Emotional prosody processing for non-native English speakers. Springer.

[bibr7-17456916221148142] Barker R. G. (1968). Ecological psychology: Concepts and methods for studying the environment of human behavior. Stanford University Press.

[bibr8-17456916221148142] Barrett L. F. (2019, October). Zombie ideas [Editorial]. APS Observer. https://www.psychologicalscience.org/observer/zombie-ideas

[bibr9-17456916221148142] Barrett L. F. Adolphs R. Martinez A. Marsella S. Pollak S. (2019). Emotional expressions reconsidered: Challenges to inferring emotion in human facial movements. Psychological Science in the Public Interest, 20, 1–68. 10.1177/1529100619832930 31313636 PMC6640856

[bibr10-17456916221148142] Bavelas J. B. (2007). Face-to-face dialogue as a micro-social context: The example of motor mimicry. In Duncan S. D. Cassell J. Levy E. T. (Eds.), Gesture and the dynamic dimension of language: Essays in honor of David McNeill (pp. 127–146). John Benjamins.

[bibr11-17456916221148142] Bavelas J. B. Black A. Chovil N. Lemery C. R. Mullett J. (1988). Form and function in motor mimicry: Topographic evidence that the primary function is communicative. Human Communication Research, 14, 275–299. 10.1111/j.1468-2958.1988.tb00158.x

[bibr12-17456916221148142] Bavelas J. B. Black A. Lemery C. R. Mullett J. (1986). “I show how you feel”: Motor mimicry as a communicative act. Journal of Personality and Social Psychology, 50, 322–329. 10.1037/0022-3514.50.2.322

[bibr13-17456916221148142] Bazant M. Z. Bush J. W. M. (2021). A guideline to limit indoor airborne transmission of COVID-19. Proceedings of the National Academy of Sciences, USA, 118(17), Article e2018995118. 10.1073/pnas.2018995118 PMC8092463 33858987

[bibr14-17456916221148142] Birdwhistell R. L. (1952). Introduction to kinesics. Foreign Service Institute.

[bibr15-17456916221148142] Birdwhistell R. L. (1970). Kinesics and context: Essays in body motion communication. University of Pennsylvania Press.

[bibr16-17456916221148142] Bogardus E. S. (1925). Measuring social distances. Journal of Applied Sociology, 9, 299–308.

[bibr17-17456916221148142] Bogardus E. S. (1926). Social distance in the city. Proceedings and Publications of the American Sociological Society, 20, 40–46.

[bibr18-17456916221148142] Bond C. F. Jr. (2008). A few can catch a liar, sometimes: Comments on Ekman and O’Sullivan (1991), as well as Ekman, O’Sullivan, and Frank (1999). Applied Cognitive Psychology, 22, 1298–1300. 10.1002/acp.1475

[bibr19-17456916221148142] Bond C. F. Jr. DePaulo B. M. (2008). Individual differences in judging deception: Accuracy and bias. Psychological Bulletin, 134, 477–492. 10.1037/0033-2909.134.4.477 18605814

[bibr20-17456916221148142] Bond C. F. Jr. Uysal A. (2007). On lie detection “wizards.” Law and Human Behavior, 31, 109–115. 10.1007/s10979-006-9016-1 17221309

[bibr21-17456916221148142] Bouchard D. (2013). The nature and origin of language. Oxford University Press.

[bibr22-17456916221148142] Brodsky S. L. Hooper N. E. Tipper D. G. Yates S. B. (1999). Attorney invasion of witness space. Law & Psychology Review, 23, 49–68.

[bibr23-17456916221148142] Brown D. E. (1991). Human universals. McGraw-Hill.

[bibr24-17456916221148142] Burgoon J. K. (2018). Microexpressions are not the best way to catch a liar. Frontiers in Psychology, 9, Article 1672. 10.3389/fpsyg.2018.01672 PMC6158306 30294288

[bibr25-17456916221148142] Burgoon J. K. Dunbar N. E. (2006). Nonverbal expressions of dominance and power in human relationships. In Manusov V. Patterson M. L. (Eds.), The SAGE handbook of nonverbal communication (p. 2). SAGE. 10.4135/9781412976152.n15

[bibr26-17456916221148142] Carissimi N. Beyan C. Murino V. (2018, May). A multi-view learning approach to deception detection. Paper presented at the 13th IEEE International Conference on Automatic Face & Gesture Recognition, Verona. 10.1109/FG.2018.00095

[bibr27-17456916221148142] Cartmill E. A. Goldin-Meadow S. (2016). Gesture. In Matsumoto D. Hwang H. C. Frank M. G. (Eds.), APA handbook of nonverbal communication (pp. 307–333). American Psychological Association.

[bibr28-17456916221148142] Celeghin A. Diano M. Bagnisa A. Viola M. Tamietto M. (2017). Basic emotions in human neuroscience: Neuroimaging and beyond. Frontiers in Psychology, 8, Article 1432. 10.1016/j.tics.2018.02.006 PMC5573709 28883803

[bibr29-17456916221148142] Chapman G. (1992). The five love languages: How to express heartfelt commitment to your mate. Northfield.

[bibr30-17456916221148142] Chivers T. (2019, July 4). Does psychology have a conflict-of-interest problem? Nature, 571, 20–23. https://media.nature.com/original/magazine-assets/d41586-019-02041-5/d41586-019-02041-5.pdf 31267062 10.1038/d41586-019-02041-5

[bibr31-17456916221148142] Cloutier J. Macrae C. N. (2007). Who or what are you? Facial orientation and person construal. European Journal of Social Psychology, 37, 1298–1309.

[bibr32-17456916221148142] Colombetti G. (2014). The feeling body: Affective science meets the enactive mind. MIT Press.

[bibr33-17456916221148142] Corballis M. C. (2014). Left brain, right brain: Facts and fantasies. PLOS Biology, 12(1), Article e1001767. 10.1371/journal.pbio.1001767 PMC3897366 24465175

[bibr34-17456916221148142] Crivelli C. Fridlund A. J. (2018). Facial displays are tools for social influence. Trends in Cognitive Sciences, 22(5), 388–399. 10.1016/j.tics.2018.02.006 29544997

[bibr35-17456916221148142] Crivelli C. Fridlund A. J. (2019). Inside-out: From basic emotions theory to the behavioral ecology view. Journal of Nonverbal Behavior, 43, 161–194. 10.1007/s10919-019-00294-2

[bibr36-17456916221148142] Crivelli C. Gendron M. (2017). Facial expressions and emotions in indigenous societies. In Fernández-Dols J. M. Russell J. A. (Eds.), The science of facial expression (pp. 497–515). Oxford University Press.

[bibr37-17456916221148142] Crivelli C. Jarillo S. Fridlund A. J. (2016). A multidisciplinary approach to research in small-scale societies: Studying emotions and facial expressions in the field. Frontiers in Psychology, 7, Article 1073. 10.3389/fpsyg.2016.01073 PMC4947591 27486420

[bibr38-17456916221148142] Crivelli C. Jarillo S. Russell J. A. Fernández-Dols J. M. (2016). Reading emotions from faces in two indigenous societies. Journal of Experimental Psychology: General, 145, 830–843. 10.1037/xge0000172 27100308

[bibr39-17456916221148142] Crivelli C. Russell J. A. Jarillo S. Fernández-Dols J. M. (2016). The fear gasping face as a threat display in a Melanesian society. Proceedings of the National Academy of Sciences, USA, 113, 12403–12407. 10.1073/pnas.1611622113 PMC5098662 27791137

[bibr40-17456916221148142] Crivelli C. Russell J. A. Jarillo S. Fernández-Dols J.-M. (2017). Recognizing spontaneous facial expressions of emotion in a small-scale society of Papua New Guinea. Emotion, 17(2), 337–347. 10.1037/emo0000236 27736108

[bibr41-17456916221148142] Darwin C. R. (1872). The expression of the emotions in man and animals. Albemarle.

[bibr42-17456916221148142] Denault V. Plusquellec P. Jupe L. M. St-Yves M. Dunbar N. E. Hartwig M. Sporer S. L. Rioux-Turcotte J. Jarry J. Walsh D. Otgaar H. Viziteu A. Talwar V. Keatley D. A. Blandón-Gitlin I. Townson C. Deslauriers-Varin N. Lilienfeld S. O. Patterson M. L. . . . van Koppen P. J. (2020). The analysis of nonverbal communication: The dangers of pseudoscience in security and justice contexts. Anuario de Psicología Jurídica, 30(1), 1–12. 10.5093/apj2019a9

[bibr43-17456916221148142] DePaulo B. M. Lindsay J. L. Malone B. E. Muhlenbruck L. Charlton K. Cooper H. (2003). Cues to deception. Psychological Bulletin, 129, 74–118. 10.1037/0033-2909.129.1.74 12555795

[bibr44-17456916221148142] Durán J. I. Fernández-Dols J. M. (2021). Do emotions result in their predicted facial expressions? A meta-analysis of studies on the link between expression and emotion. Emotion, 21(7), 1550–1569. 10.1037/emo0001015 34780241

[bibr45-17456916221148142] Durán J. I. Reisenzein R. Fernández-Dols J. M. (2017). Coherence between emotions and facial expressions: A research synthesis. In Fernández-Dols J. M. Russell J. A. (Eds.), The science of facial expression (pp. 107–129). Oxford University Press.

[bibr46-17456916221148142] Eibl-Eibesfeldt I. (1989). Human ethology. Aldine de Gruyter.

[bibr47-17456916221148142] Ekman P. (1965). Differential communication of affect by head and body cues. Journal of Personality and Social Psychology, 2, 726–735. 10.1037/h0022736 5838771

[bibr48-17456916221148142] Ekman P. (1980). The face of man: Expressions of universal emotions in a New Guinea village. Garland.

[bibr49-17456916221148142] Ekman P. (1992). An argument for basic emotions. Cognition and Emotion, 6(3–4), 169–200. 10.1080/02699939208411068

[bibr50-17456916221148142] Ekman P. (2003). Emotions revealed. Owl Books, Holt.

[bibr51-17456916221148142] Ekman P. (2009). Telling lies: Clues to deceit in the marketplace, politics, and marriage. W. W. Norton.

[bibr52-17456916221148142] Ekman P. (2017). Facial expressions. In Fernández-Dols J. M. Russell J. A. (Eds.), The science of facial expression (pp. 39–56). Oxford University Press.

[bibr53-17456916221148142] Ekman P. Cordaro D. (2011). What is meant by calling emotions basic. Emotion Review, 3(4), 364–370. 10.1177/1754073911410740

[bibr54-17456916221148142] Ekman P. Davidson R. J. Friesen W. V. (1990). The Duchenne smile: Emotional expression and brain physiology: II. Journal of Personality and Social Psychology, 58(2), 342–353. 10.1037/0022-3514.58.2.342 2319446

[bibr55-17456916221148142] Ekman P. Friesen W. V. (1969). Nonverbal behavior and cues to deception. Psychiatry, 32, 88–106. 5779090 10.1080/00332747.1969.11023575

[bibr56-17456916221148142] Ekman P. Friesen W. V. (1971). Constants across cultures in the face and emotion. Journal of Personality and Social Psychology, 17, 124–129. 10.1037/h0030377 5542557

[bibr57-17456916221148142] Ekman P. Friesen W. V. (1974a). Detecting deception from the body or face. Journal of Personality and Social Psychology, 29, 288–298. 10.1037/h0036006

[bibr58-17456916221148142] Ekman P. Friesen W. V. (1974b). Nonverbal behavior and psychopathology. In Friedman R. J. Katz M. (Eds.), The psychology of depression: Contemporary theory and research (pp. 3–31). Winston and Sons.

[bibr59-17456916221148142] Ekman P. Friesen W. V. (1975). Unmasking the face: A guide to recognizing emotions from facial clues. Prentice-Hall.

[bibr60-17456916221148142] Ekman P. Friesen W. V. (1982). Felt, false, and miserable smiles. Journal of Nonverbal Behavior, 6, 238–252. 10.1007/BF00987191

[bibr61-17456916221148142] Ekman P. O’Sullivan M. Frank M. (2008). Reply scoring and reporting: A response to Bond (2008). Applied Cognitive Psychology, 22(9), 1315–1317. 10.1002/acp.1474

[bibr62-17456916221148142] Ekman P. Sorenson E. R. Friesen W. V. (1969). Pan-cultural elements in facial displays of emotion. Science, 164(3875), 86–88. 10.1126/science.164.3875.86 5773719

[bibr63-17456916221148142] Elfenbein H. A. Beaupré M. Lévesque M. Hess U. (2007). Toward a dialect theory: Cultural differences in the expression and recognition of posed facial expressions. Emotion, 7(1), 131–146. 10.1037/1528-3542.7.1.131 17352569

[bibr64-17456916221148142] Esposito A. Jain L. C. (Eds.). (2016). Toward robotic socially believable behaving systems (Vols. 1–2). Springer.

[bibr65-17456916221148142] Ethington P. (1997, September). The intellectual construction of “social distance”: Toward a recovery of Georg Simmel’s social geometry. Cybergeo: European Journal of Geography. 10.4000/cybergeo.227

[bibr66-17456916221148142] Fast J. (1970). Body language. Simon & Schuster.

[bibr67-17456916221148142] Felipe N. J. Sommer R. (1966). Invasion of personal space. Social Problems, 14, 206–214.

[bibr68-17456916221148142] Fernández-Dols J. M. Carrera P. (2010). *Le bon dieu est dans le detail*: Is smiling the recognition of happiness? Behavioral and Brain Sciences, 33(6), 446–447. 10.1017/S0140525X10001512

[bibr69-17456916221148142] Floegel M. Fuchs S. Kell C. A. (2020). Differential contributions of the two cerebral hemispheres to temporal and spectral speech feedback control. Nature Communications, 11, Article 2839. 10.1038/s41467-020-16743-2 PMC7275068 32503986

[bibr70-17456916221148142] Freud S. (1915). The psychopathology of everyday life ( Brill A. A. , Trans.). Macmillan. (Original work published 1901)

[bibr71-17456916221148142] Fridlund A. J. (1991a). Evolution and facial action in reflex, social motive, and paralanguage. Biological Psychology, 32, 3–100. 10.1016/0301-0511(91)90003-Y 1742403

[bibr72-17456916221148142] Fridlund A. J. (1991b). Sociality of solitary smiling: Potentiation by an implicit audience. Journal of Personality and Social Psychology, 60(2), 229–240. 10.1037/0022-3514.60.2.229

[bibr73-17456916221148142] Fridlund A. J. (1994). Human facial expression: An evolutionary view. Academic Press.

[bibr74-17456916221148142] Fridlund A. J. (2017). The behavioral ecology view of facial displays, 25 years later. In Fernández-Dols J. M. Russell J. A. (Eds.), The science of facial expression (pp. 77–92). Oxford University Press.

[bibr75-17456916221148142] Fridlund A. J. (2021). Do faces lie? Ted Bundy and the smiles of strangers. In Liberman C. J. Wrench J. S. (Eds.), Casing nonverbal communication (pp. 109–125). Kendall Hunt.

[bibr76-17456916221148142] Fridlund A. J. Duchaine B. (1996). “Facial expressions of emotion” and the delusion of the hermetic self. In Harré R. Parrott W. G. (Eds.), The emotions (pp. 259–284). Cambridge University Press.

[bibr77-17456916221148142] Fridlund A. J. Hatfield M. E. Cottam G. L. Fowler S. C. (1986). Anxiety and striate-muscle activation: Evidence from electromyographic pattern analysis. Journal of Abnormal Psychology, 95, 228–236. 10.1037//0021-843x.95.3.228 3745644

[bibr78-17456916221148142] Fridlund A. J. Russell J. A. (in press). Evolution, emotion and facial behavior: A 21st-century view. In Al-Shawaf L. Shackelford T. (Eds.), The Oxford handbook of evolution and the emotions. Oxford University Press.

[bibr79-17456916221148142] Gendron M. Crivelli C. Barrett L. F. (2018). Universality reconsidered: Diversity in making meaning of facial expressions. Current Directions in Psychological Science, 27, 211–219. 10.1177/0963721417746794 30166776 PMC6099968

[bibr80-17456916221148142] Gendron M. Hoemann K. Crittenden A. N. Mangola S. M. Ruark G. A. Barrett L. F. (2020). Emotion perception in Hadza hunter-gatherers. Scientific Reports, 10(1), 3867. 10.1038/s41598-020-60257-2 32123191 PMC7051983

[bibr81-17456916221148142] Gendron M. Roberson D. Barrett L. F. (2015). Cultural variation in emotion perception is real: A response to Sauter et al. Psychological Science, 26, 357–359. 10.1177/0956797614566659 25608863 PMC5497728

[bibr82-17456916221148142] Gendron M. Roberson D. van der Vyver J. M. Barrett L. F. (2014). Perceptions of emotion from facial expressions are not culturally universal: Evidence from a remote culture. Emotion, 14(2), 251–262. 10.1037/a0036052 24708506 PMC4752367

[bibr83-17456916221148142] Girard J. M. Cohn J. F. Yin L. Morency L.-P. (2021). Reconsidering the Duchenne smile: Formalizing and testing hypotheses about eye constriction and positive emotion. Affective Science, 2, 32–47. 10.1007/s42761-020-00030-w 34337430 PMC8317937

[bibr84-17456916221148142] Gladwell M. (2005). Blink: The power of thinking without thinking. Penguin Books.

[bibr85-17456916221148142] Gladwell M. (2019). Talking to strangers. Penguin Books.

[bibr86-17456916221148142] Goffman E. (1963). Behavior in public places. The Free Press.

[bibr87-17456916221148142] Goffman E. (1971). The presentation of self in everyday life. Pelican Books.

[bibr88-17456916221148142] Goldin-Meadow S. Alibali M. W. (2013). Gesture’s role in speaking, learning, and creating language. Annual Review of Psychology, 123, 448–453. 10.1146/annurev-psych-113011-143802 PMC3642279 22830562

[bibr89-17456916221148142] Gosling S. D. Gaddis S. Vazire S. (2008). First impressions based on the environments we create and inhabit. In Ambady N. Skowronski J. J. (Eds.), First impressions (pp. 334–356). Guilford Press.

[bibr90-17456916221148142] Haamer R. E. Rusadze E. Lüsi I. Ahmed T. Escalera S. Anbarjafari G. (2018). Review on emotion recognition databases. In Anbarjafari G. Escalera S. (Eds.), Human-robot interaction: Theory and application (pp. 39–63). IntechOpen. 10.5772/intechopen.72748

[bibr91-17456916221148142] Haggard E. A. Isaacs K. S. (1966). Micro-momentary facial expressions as indicators of ego mechanisms in psychotherapy. In Gottschalk L. A. Auerbach A. H. (Eds.), Methods of research in psychotherapy (pp. 154–165). Appleton-Century-Crofts.

[bibr92-17456916221148142] Hall E. T. (1959). The silent language. Doubleday.

[bibr93-17456916221148142] Hall E. T. (1966). The hidden dimension. Doubleday.

[bibr94-17456916221148142] Hall J. A. Gunnery S. D. (2013). Gender differences in nonverbal communication. In Hall J. A. Knapp M. L. (Eds.), Nonverbal communication (pp. 639–670). De Gruyter Mouton. 10.1515/9783110238150.639

[bibr95-17456916221148142] Hall J. A. Knapp M. L. (Eds.). (2013). Nonverbal communication. De Gruyter Mouton.

[bibr96-17456916221148142] Hartwig M. Bond C. F. Jr. (2011). Why do lie-catchers fail? A lens model meta-analysis of human lie judgments. Psychological Bulletin, 137, 643–659. 10.1037/a0023589 21707129

[bibr97-17456916221148142] Hayduk L. A. (1983). Personal space: Where we now stand. Psychological Bulletin, 94(2), 293–335. 10.1037/0033-2909.94.2.293

[bibr98-17456916221148142] Hazlett R. L. McLeod D. R. Hoehn-Saric R. (1994). Muscle tension in generalized anxiety disorder: Elevated muscle tonus or agitated movement? Psychophysiology, 31, 189–195. 10.1111/j.1469-8986.1994.tb01039.x 8153255

[bibr99-17456916221148142] Henrich J. Heine S. J. Norenzayan A. (2010). The weirdest people in the world? Behavioral and Brain Sciences, 33(2–3), 61–83. 10.1017/S0140525X0999152X 20550733

[bibr100-17456916221148142] Hickok G. Bellugi U. Klima E. S. (2001). Sign language in the brain. Scientific American, 284(6), 58–65. 10.1038/scientificamerican0601-58 11396343

[bibr101-17456916221148142] Hinde R. A. (1985). Was “the expression of the emotions” a misleading phrase? Animal Behaviour, 33(3), 985–992. 10.1016/S0003-3472(85)80032-4

[bibr102-17456916221148142] Houston P. Floyd M. Carnicero S. Tennant D. (2012). Spy the lie: Former CIA officers teach you how to detect deception. St. Martin’s.

[bibr103-17456916221148142] Inbau F. E. Reid J. E. Buckley J. P. Jayne B. C. (2013). Criminal interrogation and confessions. Jones and Bartlett.

[bibr104-17456916221148142] Ioannidis J. (2012). Why science is not necessarily self-correcting. Perspectives on Psychological Science, 7, 645–654. 10.1177/1745691612464056 26168125

[bibr105-17456916221148142] Jack R. E. Garrod O. G. B. Yu H. Caldara R. Schyns P. G. (2012). Facial expressions of emotion are not culturally universal. Proceedings of the National Academy of Sciences, USA, 109, 7241–7244. 10.1073/pnas.1200155109 PMC3358835 22509011

[bibr106-17456916221148142] Jordan S. Brimbal L. Wallace D. B. Kassin S. Hartwig M. Street C. N. H. (2019). A test of the micro-expressions training tool: Does it improve lie detection? Journal of Investigative Psychology and Offender Profiling, 16, 222–235. 10.1002/jip.1532

[bibr107-17456916221148142] Jung S. Proske M. Kahl K. G. Krüger T. H. C. Wollmer M. A. (2016). The New Hamburg-Hannover Agitation Scale in clinical samples: Manifestation and differences of agitation in depression, anxiety, and borderline personality disorder. Psychopathology, 49, 420–428. 10.1159/000451029 27846624

[bibr108-17456916221148142] Jupe L. M. Denault V. (2019). Science or pseudoscience? A distinction that matters for police officers, lawyers and judges. Psychiatry, Psychology and Law, 26(5), 753–765. 10.1080/13218719.2019.1618755 PMC6896483 31984109

[bibr109-17456916221148142] Jupe L. M. Keatley D. A. (2020). Airport artificial intelligence can detect deception: Or am I lying? Security Journal, 33, 622–635. 10.1057/s41284-019-00204-7

[bibr110-17456916221148142] Kagan J. (2007). What is an emotion? History, measures, and meanings. Yale University Press.

[bibr111-17456916221148142] Keltner D. Oatley K. (2022). Social functions of emotions in life and imaginative culture. Evolutionary Studies in Imaginative Culture, 6, 1–20. 10.26613/esic.6.1.263

[bibr112-17456916221148142] Key M. R. (1962). Gestures and responses: A preliminary study among some Indian tribes of Bolivia. Studies in Linguistics, 16, 92–99.

[bibr113-17456916221148142] Knapp M. L. (2006). An historical overview of nonverbal research. In Manusov V. Patterson M. L. (Eds.), The SAGE handbook of nonverbal communication (pp. 3–19). SAGE.

[bibr114-17456916221148142] Knapp M. L. Hall J. A. Horgan T. G. (Eds.). (2012). Nonverbal communication in human interaction (8th ed.). Wadsworth.

[bibr115-17456916221148142] Krauss R. M. (1998). Why do we gesture when we speak? Current Directions in Psychological Science, 7, 54–60. https://www.jstor.org/stable/20182502

[bibr116-17456916221148142] Krebs J. R. Davies N. B. (Eds.). (1978). Behavioural ecology: An evolutionary approach. Blackwell.

[bibr117-17456916221148142] Krebs J. R. Davies N. B. (Eds.). (1987). An introduction to behavioural ecology (2nd ed.). Blackwell.

[bibr118-17456916221148142] Krebs J. R. Dawkins R. (1984). Animal signals: Mind-reading and manipulation. In Krebs J. R. Davies N. B. (Eds.), Behavioural ecology (2nd ed., pp. 380–402). Blackwell.

[bibr119-17456916221148142] Krumhuber E. G. Kappas A. (2022). More what Duchenne smiles do, less what they express. Perspectives on Psychological Science. Advance online publication. 10.1177/17456916211071083 35712993

[bibr120-17456916221148142] Krumhuber E. G. Manstead A. S. R. (2009). Can Duchenne smiles be feigned? New evidence on felt and false smiles. Emotion, 9, 807–820. 10.1037/a0017844 20001124

[bibr121-17456916221148142] Lakin J. L. (2013). Behavioral mimicry and interpersonal synchrony. In Hall J. A. Knapp M. L. (Eds.), Nonverbal communication (pp. 539–576). De Gruyter Mouton. 10.1515/9783110238150.539

[bibr122-17456916221148142] Levinson S. C. (2003). Space in language and cognition. Cambridge University Press.

[bibr123-17456916221148142] Leys R. (2017). The ascent of affect: Genealogy and critique. University of Chicago Press.

[bibr124-17456916221148142] Li X. Hong X. Moilanen A. Huang X. Pfister T. Zhao G. Pietikäinen M. (2018). Towards reading hidden emotions: A comparative study of spontaneous micro-expression spotting and recognition methods. IEEE Transactions on Affective Computing, 9(4), 563–577. 10.1109/TAFFC.2017.2667642

[bibr125-17456916221148142] Liljenquist K. A. Zhong C.-B. Galinsky A. D. (2010). The smell of virtue: Clean scents promote reciprocity and charity. Psychological Science, 21, 381–383. 10.1177/0956797610361426 20424074

[bibr126-17456916221148142] Lin L. Ginns P. Wang T. Zhang P. (2020). Using a pedagogical agent to deliver conversational style instruction: What benefits can you obtain? Computers & Education, 143, Article 103658. 10.1016/j.compedu.2019.103658

[bibr127-17456916221148142] Lindquist K. A. Jackson J. C. Leshin J. Satpute A. B. Gendron M. (2022). The cultural evolution of emotion. Nature Reviews Psychology. Advance online publication. 10.1038/s44159-022-00105-4

[bibr128-17456916221148142] Lindquist K. A. Wager T. D. Kober H. Bliss-Moreau E. Barrett L. F. (2012). The brain basis of emotion: A meta-analytic review. Behavioral and Brain Sciences, 35(3), 121–143. 10.1017/S0140525X11000446 22617651 PMC4329228

[bibr129-17456916221148142] Lloyd D. M. Coates A. Knopp J. Oram S. Rowbotham S. (2009). Don’t stand so close to me: The effect of auditory input on interpersonal space. Perception, 38, 617–620. 10.1068/p6317 19522329

[bibr130-17456916221148142] Lorenz K. Z. (1967). Studies on animal and human behavior (Vols. 1 and 2). Harvard University Press.

[bibr131-17456916221148142] Lycan W. G. (2019). Philosophy of language (3rd ed.). Routledge.

[bibr132-17456916221148142] MacKay D. M. (1972). Formal analysis of communication processes. In Hinde R. A. (Ed.), Nonverbal communication (pp. 3–25). Cambridge University Press.

[bibr133-17456916221148142] Malinowski B. (1922). Argonauts of the Western Pacific. Routledge & Kegan Paul.

[bibr134-17456916221148142] Manusov V. Patterson M. L. (Eds.). (2006). SAGE handbook of nonverbal communication. SAGE.

[bibr135-17456916221148142] Marler P. R. Duffy A. Pickert R . (1986a). Vocal communication in the domestic chicken: I. Does a sender communicate information about the quality of a food referent to a receiver? Animal Behaviour, 34, 188–193. 10.1006/ccog.1993.1022

[bibr136-17456916221148142] Marler P. R. Duffy A. Pickert R. (1986b). Vocal communication in the domestic chicken: II. Is a sender sensitive to the presence and nature of a receiver? Animal Behaviour, 34, 194–198. 10.1016/0003-3472(86)90023-0

[bibr137-17456916221148142] Marquardt G. Cross E. S. de Sousa A. A. Edelstein E. Farnè A. Leszczynski M. Patterson M. Quadflieg S. (2015). There or not there? A multidisciplinary review and research agenda on the impact of transparent barriers on human perception, action, and social behavior. Frontiers in Psychology, 6, Article 1381. 10.3389/fpsyg.2015.01381 PMC4569749 26441756

[bibr138-17456916221148142] Martin J. Rychlowska M. Wood A. Niedenthal P. (2017). Smiles as multipurpose social signals. Trends in Cognitive Science, 11, 864–877. 10.1016/j.tics.2017.08.007 28964655

[bibr139-17456916221148142] Matsumoto D. Hwang H. C. Frank M. G. (Eds.). (2016). APA handbook of nonverbal communication. American Psychological Association.

[bibr140-17456916221148142] Matsumoto D. Hwang H. S. (2018). Microexpressions differentiate truths from lies about future malicious intent. Frontiers in Psychology, 9, Article 2545. 10.3389/fpsyg.2018.02545 PMC6305322 30618966

[bibr141-17456916221148142] Matsumoto D. Lee M. (1993). Consciousness, volition, and the neuropsychology of facial expressions of emotion. Consciousness and Cognition, 2, 237–254. 10.1006/ccog.1993.1022

[bibr142-17456916221148142] Maynard Smith J . (1982). Evolution and the theory of games. Cambridge University Press.

[bibr143-17456916221148142] McArthur J. A. (2016). Digital proxemics. Peter Lang.

[bibr144-17456916221148142] McDuff D. A. Mahmoud M. Mavadati M. Amr J. Turcot J. el Kaliouby R. (2016). AFFDEX SDK: A cross-platform real-time multi-face expression recognition toolkit. In Proceedings of the 2016 CHI Conference Extended Abstracts on Human Factors in Computing Systems (pp. 3723–3726). Association for Computing Machinery.

[bibr145-17456916221148142] McNeill D. (1985). So you think gestures are nonverbal? Psychological Bulletin, 92, 350–371. 10.1037/0033-295X.92.3.350

[bibr146-17456916221148142] Medin D. L. (2017). Psychological science as a complex system: Report card. Perspectives on Psychological Science, 12(4), 669–674. 10.1177/1745691616687746 28727964

[bibr147-17456916221148142] Medin D. L. Ojalehto B. Marin A. Bang M. (2017). Systems of (non-)diversity. Nature Human Behaviour, 1(5), Article 0088. 10.1038/s41562-017-0088

[bibr148-17456916221148142] Merton R. K. (1948). The self-fulfilling prophecy. Antioch Review, 8, 193–210. 10.2307/4609267

[bibr149-17456916221148142] Meyer C. (2014). Gestures in West Africa: Wolof. In Müller C. Cienki A. Fricke E. Ladewig S. H. McNeill D. Bressem J. (Eds.), Body - language - communication: An international handbook on multimodality in human interaction (pp. 1169–1175). De Gruyter Mouton.

[bibr150-17456916221148142] Montagu J. (1994). The expression of the passions. Yale University Press.

[bibr151-17456916221148142] Morris J. Frith C. Perrett D. Rowland D. Young A. W. Calder A. J. Dolan R. J. (1996). A differential neural response in the human amygdala to fearful and happy facial expressions. Nature, 383, 812–815. 10.1038/383812a0 8893004

[bibr152-17456916221148142] Navarro J. Karlins M. (2008). What every body is saying: An ex-FBI agent’s guide to speed-reading people. Collins.

[bibr153-17456916221148142] Nelson N. Russell J. A. (2013). Universality revisited. Emotion Review, 5, 8–15. 10.1177/1754073912457227

[bibr154-17456916221148142] Nickerson R. S. (1998). Confirmation bias: A ubiquitous phenomenon in many guises. Review of General Psychology, 2(2), 175–220. 10.1037/1089-2680.2.2.175

[bibr155-17456916221148142] Nierenberg G. I. Calero H. H. Grayson G. (2010). How to read a person like a book. SquareOne Publishers.

[bibr156-17456916221148142] Oishi S. Graham J. (2010). Social ecology: Lost and found in psychological science. Perspectives on Psychological Science, 5(4), 356–377. 10.1177/1745691610374588 26162183

[bibr157-17456916221148142] Open Science Collaboration. (2015). Estimating the reproducibility of psychological science. Science, 349(6251), 1–8. 10.1126/science.aac4716 26315443

[bibr158-17456916221148142] Ortony A. (2021). Are all “basic emotions” emotions? A problem for the (basic) emotions construct. Perspectives on Psychological Science, 17(1), 41–61. 10.1177/1745691620985415 34264141

[bibr159-17456916221148142] O’Sullivan M. Ekman P. Friesen W. V. (1988). The effect of comparisons on detecting deceit. Journal of Nonverbal Behavior, 12, 203–215.

[bibr160-17456916221148142] Patterson M. L. (1973). Compensation in nonverbal immediacy behaviors: A review. Sociometry, 36, 237–252. 10.2307/2786569

[bibr161-17456916221148142] Patterson M. L. (1975). Personal space: Time to burst the bubble? Man-Environment Systems, 5(2), 67.

[bibr162-17456916221148142] Patterson M. L. (1976). An arousal model for interpersonal intimacy. Psychological Review, 83(3), 235–245. 10.1080/01973530802208816

[bibr163-17456916221148142] Patterson M. L. (1978). The role of space in social interaction. In Siegman A. Feldstein S. (Eds.), Nonverbal behavior and communication (pp. 265–290). Erlbaum.

[bibr164-17456916221148142] Patterson M. L. (1983). Nonverbal behavior: A functional perspective. Springer-Verlag.

[bibr165-17456916221148142] Patterson M. L. (1995). A parallel process model of nonverbal communication. Journal of Nonverbal Behavior, 19, 3–29. 10.1007/BF02173410

[bibr166-17456916221148142] Patterson M. L. (2008). Back to social behavior: Mining the mundane. Basic and Applied Social Psychology, 30, 93–101. 10.2307/2786354

[bibr167-17456916221148142] Patterson M. L. (2011). More than words: The power of nonverbal communication. Aresta.

[bibr168-17456916221148142] Patterson M. L. (2019). A systems model of dyadic nonverbal interaction. Journal of Nonverbal Behavior, 43, 111–132. 10.1177/1529100619832930

[bibr169-17456916221148142] Patterson M. L. (2021). The power of the setting in nonverbal communication. In Liberman C. J. Wrench J. S. (Eds.), Casing nonverbal communication (pp. 71–81). Kendall Hunt.

[bibr170-17456916221148142] Patterson M. L. Mullens S. Romano J. (1971). Compensatory reactions to spatial intrusion. Sociometry, 34, 114–121. 10.2307/2786354

[bibr171-17456916221148142] Patterson M. L. Quadflieg S. (2016). The physical environment and nonverbal communication. In Matsumoto D. Hwang H. C. Frank M. G. (Eds.), APA handbook of nonverbal communication (pp. 189–220). American Psychological Association.

[bibr172-17456916221148142] Philippot P. Feldman R. S. Coats E. J. (Eds.). (2003). Nonverbal behavior in clinical settings. Oxford University Press.

[bibr173-17456916221148142] Porter S. ten Brinke L. (2008). Reading between the lies: Identifying concealed and falsified emotions in universal facial expressions. Psychological Science, 19, 508–514. 10.1111/j.1467-9280.2008.02116.x 18466413

[bibr174-17456916221148142] Porter S. ten Brinke L. Wallace B. (2012). Secrets and lies: Involuntary leakage in deceptive facial expressions as a function of emotional intensity. Journal of Nonverbal Behavior, 36, 23–37. 10.1007/s10919-011-0120-7

[bibr175-17456916221148142] Rai T. S. Fiske A. (2010). ODD (observation-and description-deprived) psychological research. Behavioral and Brain Sciences, 33, 106–107. 10.1007/BF02173410 20546657

[bibr176-17456916221148142] Rapoport A. (1982). The meaning of the built environment: A nonverbal communication approach. University of Arizona Press.

[bibr177-17456916221148142] Reiter S. (2014). Gestures in South American indigenous cultures. In Müller C. Cienki A. Fricke E. Ladewig S. H. McNeill D. Bressem J. (Eds.), Body – language – communication: An international handbook on multimodality in human interaction (pp. 1182–1193). De Gruyter Mouton.

[bibr178-17456916221148142] Richerson P. J. Boyd R. (2005). Not by genes alone: How culture transformed human evolution. University of Chicago Press.

[bibr179-17456916221148142] Russell J. A. (1994). Is there universal recognition of emotion from facial expression? A review of the cross-cultural studies. Psychological Bulletin, 115, 102–141. 10.1037/0033-2909.115.1.102 8202574

[bibr180-17456916221148142] Russell J. A. (1995). Facial expressions of emotion: What lies beyond minimal universality? Psychological Bulletin, 188(3), 379–391. 10.1037/0033-2909.118.3.379 7501742

[bibr181-17456916221148142] Russell J. A. (2017). Toward a broader perspective on facial expressions: Moving on from basic emotion theory. In Fernández-Dols J. M. Russell J. A. (Eds.), The science of facial expression (pp. 93–107). Oxford University Press.

[bibr182-17456916221148142] Scarantino A. (2017). How to do things with emotional expressions: The theory of affective pragmatics. Psychological Inquiry, 28(2–3), 165–185. 10.1080/1047840X.2017.1328951

[bibr183-17456916221148142] Scarantino A. Hareli S. Hess U. (2021). Emotional expressions as appeals to recipients. Emotion. Advance online publication. 10.1037/emo0001023 34766791

[bibr184-17456916221148142] Schaeffer G. H. Patterson M. L. (1980). Intimacy, arousal, and small group crowding. Journal of Personality and Social Psychology, 38(2), 283–290. 10.1037/0022-3514.38.2.283 7373513

[bibr185-17456916221148142] Schützwohl A. Reisenzein R. (2012). Facial expressions in response to a highly surprising event exceeding the field of vision: A test of Darwin’s theory of surprise. Evolution and Human Behavior, 33, 657–664. 10.1016/j.evolhumbehav.2012.04.003

[bibr186-17456916221148142] Shariff A. F. Tracy J. L. (2011). What are emotion expressions for? Current Directions in Psychological Science, 20(6), 395–399. 10.1177/0963721411424739

[bibr187-17456916221148142] Simmel G. (1908). Soziologie. Duncker & Humblot.

[bibr188-17456916221148142] Smith W. J. (1977). The behavior of communicating. Harvard University Press.

[bibr189-17456916221148142] Snyder P. J. Kaufman R. Harrison J. Maruf P. (2010). Charles Darwin’s emotional expression “experiment” and his contribution to modern neuropharmacology. Journal of the History of the Neurosciences, 19, 158–170. 10.1080/09647040903506679 20446159

[bibr190-17456916221148142] Sommer R. (1961). Leadership and group geography. Sociometry, 24, 99–110. 10.2307/2785932

[bibr191-17456916221148142] Sommer R. (1969). Personal space: The behavioral basis of design. Prentice-Hall.

[bibr192-17456916221148142] Sommer R. (2002). Personal space in a digital age. In Bechtel R. B. Churchman A. (Eds.), Handbook of environmental psychology (pp. 647–660). John Wiley.

[bibr193-17456916221148142] Sternberg R. J. Kostić A. (Eds.). (2020). Social intelligence and nonverbal communication. Palgrave Macmillan.

[bibr194-17456916221148142] ten Brinke K. Porter S . (2012). Cry me a river: Identifying the behavioral consequences of extremely high-stakes interpersonal deception. Law and Human Behavior, 36(6), 469–477. 10.1037/h0093929 23205594

[bibr195-17456916221148142] ten Brinke L. Macdonald S. Porter S. O’Connor B . (2012). Crocodile tears: Facial, verbal and body language behaviours associated with genuine and fabricated remorse. Law and Human Behavior, 36(1), 51–59. 10.1037/h0093950 22471385

[bibr196-17456916221148142] Tinbergen N. (1953). Social behavior in animals. Chapman and Hall.

[bibr197-17456916221148142] Tomkins S. S. McCarter R. (1964). What and where are the primary affects? Some evidence for a theory. Perceptual and Motor Skills, 18, 119–158. 10.2466/pms.1964.18.1.119 14116322

[bibr198-17456916221148142] Tooby J. Cosmides L. (1990). The past explains the present: Emotional adaptations and the structure of ancestral environments. Ethology & Sociobiology, 11(4–5), 375–424. 10.1016/0162-3095(90)90017-Z

[bibr199-17456916221148142] Tracy J. L. (2014). An evolutionary approach to understanding distinct emotions. Emotion Review, 6, 308–312. 10.2466/pms.1964.18.1.119

[bibr200-17456916221148142] Trivers R. (2011). The folly of fools: The logic of deceit and self-deception in human life. Basic Books.

[bibr201-17456916221148142] U.S. Government Accountability Office. (2013). Aviation security: TSA should limit future funding for behavior detection activities (Report to Congressional Requesters GAO-14-159). https://www.gao.gov/products/GAO-14-159

[bibr202-17456916221148142] Vrij A. Hartwig M. Granhag P. A. (2019). Reading lies: Nonverbal communication and deception. Annual Review of Psychology, 70, 295–317. 10.1146/annurev-psych-010418-103135 30609913

[bibr203-17456916221148142] Vrij A. Mann S. Fisher R. (2006). An empirical test of the Behaviour Analysis Interview. Law and Human Behavior, 30, 329–345. 10.1007/s10979-006-9014-3 16718581

[bibr204-17456916221148142] Watzlawick P. Beavin J. (1967). Some formal aspects of communication. American Behavioral Scientist, 10, 4–8. 10.1177/0002764201000802

[bibr205-17456916221148142] Webb E. Campbell D. Schwartz R. Sechrest L. (1966). Unobtrusive measures: Nonreactive research in the social sciences. Rand McNally.

[bibr206-17456916221148142] Wheeler S. C. DeMarree K. G. (2009). Multiple mechanisms of prime-to-behavior effects. Social and Personality Compass, 3/4, 566–581. https://doi.org/10.1111.j.1751-9004.2009.00187.x

[bibr207-17456916221148142] Wicker A. W. (1979). An introduction to ecological psychology. Brooks/Cole.

[bibr208-17456916221148142] Wiener M. Devoe S. Rubinow S. Geller J. (1972). Nonverbal behavior and nonverbal communication. Psychological Review, 79, 185–214. 10.1037/h0032710

[bibr209-17456916221148142] Winter J. Currier C. (2015, March 27). TSA’s secret behavior checklist to spot terrorists. The Intercept. https://theintercept.com/2015/03/27/revealed-tsas-closely-held-behavior-checklist-spot-terrorists/

[bibr210-17456916221148142] Wundt W. (1894). Lectures on human and animal psychology. Swan Sonnenschein.

